# Characterization of data-driven clusters in diabetes-free adults and their utility for risk stratification of type 2 diabetes

**DOI:** 10.1186/s12916-022-02551-6

**Published:** 2022-10-18

**Authors:** Diego Yacamán Méndez, Minhao Zhou, Ylva Trolle Lagerros, Donaji V. Gómez Velasco, Per Tynelius, Hrafnhildur Gudjonsdottir, Antonio Ponce de Leon, Katarina Eeg-Olofsson, Claes-Göran Östenson, Boel Brynedal, Carlos A. Aguilar Salinas, David Ebbevi, Anton Lager

**Affiliations:** 1 Department of Global Public Health, Karolinska Institutet, SE-171 77 Stockholm, Sweden; 2 Center for Epidemiology and Community Medicine (CES), Stockholm Health Care Services, Stockholm, Sweden; 3 Obesity Center, Academic Specialist Center, Stockholm Health Care Services, Stockholm, Sweden; 4 Clinical Epidemiology Division, Department of Medicine, Karolinska Institutet, Stockholm, Sweden; 5 Unidad de Investigación de Enfermedades Metabólicas, Instituto Nacional de Ciencias Médicas y Nutrición “Salvador Zubirán”, Mexico City, Mexico; 6 Department of Medicine, Sahlgrenska Academy, University of Gothenburg, Gothenburg, Sweden; 7 Department of Molecular Medicine and Surgery, Karolinska Institutet, Stockholm, Sweden

**Keywords:** Precision medicine, Data-driven analysis, Type 2 diabetes, Prevention, Public health, Epidemiology

## Abstract

**Background:**

The prevention of type 2 diabetes is challenging due to the variable effects of risk factors at an individual level. Data-driven methods could be useful to detect more homogeneous groups based on risk factor variability. The aim of this study was to derive characteristic phenotypes using cluster analysis of common risk factors and to assess their utility to stratify the risk of type 2 diabetes.

**Methods:**

Data on 7317 diabetes-free adults from Sweden were used in the main analysis and on 2332 diabetes-free adults from Mexico for external validation. Clusters were based on sex, family history of diabetes, educational attainment, fasting blood glucose and insulin levels, estimated insulin resistance and β-cell function, systolic and diastolic blood pressure, and BMI. The risk of type 2 diabetes was assessed using Cox proportional hazards models. The predictive accuracy and long-term stability of the clusters were then compared to different definitions of prediabetes.

**Results:**

Six risk phenotypes were identified independently in both cohorts: very low-risk (VLR), low-risk low β-cell function (LRLB), low-risk high β-cell function (LRHB), high-risk high blood pressure (HRHBP), high-risk β-cell failure (HRBF), and high-risk insulin-resistant (HRIR). Compared to the LRHB cluster, the VLR and LRLB clusters showed a lower risk, while the HRHBP, HRBF, and HRIR clusters showed a higher risk of developing type 2 diabetes. The high-risk clusters, as a group, had a better predictive accuracy than prediabetes and adequate stability after 20 years.

**Conclusions:**

Phenotypes derived using cluster analysis were useful in stratifying the risk of type 2 diabetes among diabetes-free adults in two independent cohorts. These results could be used to develop more precise public health interventions.

**Supplementary Information:**

The online version contains supplementary material available at 10.1186/s12916-022-02551-6.

## Background

Type 2 diabetes is one of the most common causes of mortality, disability, and health expenditure worldwide [1, 2]. During recent decades, the incidence of type 2 diabetes has increased or, at best, remained stable, while its prevalence and overall burden continue to increase [3].

Heterogeneity in the pathogenesis of type 2 diabetes represents a challenge for disease prevention. For example, although overweight and obesity are relatively strong risk factors for type 2 diabetes, most individuals with a high BMI do not develop type 2 diabetes [4]. Similarly, despite prediabetes is commonly used to identify individuals at high risk of type 2 diabetes, its practical utility is still questioned. Some studies estimate that around 70% of people with prediabetes will develop type 2 diabetes during their lifetime [5], while a recent systematic review reported that regression to normal glucose levels ranged between 17 and 59% even after over 10 years, making it difficult to accurately stratify individual’s risk for type 2 diabetes [6].

Furthermore, risk stratification based on a single factor, such as glucose levels, ignores the complex pathophysiology of type 2 diabetes [7]. Recently, the World Health Organization (WHO) highlighted the need to better understand the pathophysiological heterogeneity of type 2 diabetes to improve surveillance, prevention, and treatment.

Precision medicine aims to characterize more homogeneous subpopulations based on the individuals’ biological, environmental, and social characteristics [8–10]. When extended to public health, precision prevention or precision public health aims to identify homogeneous subgroups of individuals that could lead to the development of targeted interventions for disease prevention [11, 12].

Recent studies have employed data-driven analytical methods to identify distinctive subgroups of individuals with type 2 diabetes or prediabetes and reported associations with disease progression and complications [13–15]. Given that common risk factors for type 2 diabetes vary on the individual level, we hypothesized that such methods may also be useful in identifying characteristic risk profiles before the onset of type 2 diabetes among the general population [16, 17].

The aim of this study was to determine whether cluster analysis could be used to identify homogeneous subgroups of diabetes-free adults based on the heterogeneity of known risk factors for type 2 diabetes, and asses their clinical utility to stratify the risk of type 2 diabetes compared to that of prediabetes.

## Methods

### Study samples

In this study, two independent cohorts were used. Data from the Stockholm Diabetes Prevention Program (SDPP) were used in the main analysis, while data from the Metabolic Syndrome Cohort (MSC) in Mexico were used as a validation dataset (see Additional file 1: Table S1).

#### The Stockholm Diabetes Prevention Program

Detailed information on the SDPP cohort has been published previously [18]. In summary, all habitants of five municipalities of Stockholm who were born in Sweden between 1938 and 1961 were invited by letter to participate in SDPP. The clinical subsample included in this study was created by inviting diabetes-free individuals with a family history of type 2 diabetes, women with a history of gestational diabetes, and matched controls to take part in a clinical examination including questionnaires, anthropometric and blood pressure measurements, and blood sample collection.

In total, 7948 individuals participated in the baseline examination of the clinical subsample between 1992 and 1998. The second follow-up, between 2002 and 2006, consisted of 5612 participants, and the third follow-up, between 2014 and 2017, included 4297 individuals. All baseline participants were also followed up regarding a diagnosis of type 2 diabetes by individual linkage to the clinical inpatient and outpatient registers of Region Stockholm (VAL) and the National Diabetes Registry of Sweden (NDR).

For this study, 516 (6.5%) individuals were excluded due to missing information or extreme values of any of the variables used to perform the cluster analysis and 115 (1.4%) due to a diagnosis of diabetes during the baseline examination. The resulting study sample included 7317 participants (Additional file 2: Fig. S1).

#### The Metabolic Syndrome Cohort

Detailed information on the MSC has been published previously [19]. In summary, this was a prospective cohort of 9637 diabetes-free individuals, resident in central Mexico, born in Mexico, and aged 20 years or older. Baseline examinations were performed between 2006 and 2009 at the participants’ workplace, home, or during a visit to a primary health center and included a comprehensive medical history, anthropometric and blood pressure measurements, blood sample collection, and standardized questionnaires. After 3 years (± 6 months), all participants were contacted and invited to a follow-up examination, which 6144 individuals attended (80.7%).

For this study, 1839 (19.1%) individuals younger than 30 years of age or older than 60 years at baseline were removed to ensure comparability with SDPP and to minimize the risk of including cases of type 1 diabetes or other forms of diabetes. A further 3966 (40.5%) were removed due to missing information or extreme values of variables used in the cluster analysis and 1500 (15.6%) due to loss at follow-up. The final MSC study sample thus included 2332 participants (Additional file 2: Fig. S2).

### Variables

#### Type 2 diabetes

The incidence of type 2 diabetes in the SDPP study sample was determined using oral glucose tolerance tests (OGTT) (fasting plasma glucose ≥ 7.0 mmol/L or 2-h post-load plasma glucose ≥ 11.0 mmol/L) [20], data from the VAL or NDR registers (ICD-10 code E11), or self-reported type 2 diabetes in the study questionnaires. Cases of type 1 diabetes or LADA diabetes were distinguished and excluded using the VAL and NDR registers. The OGTT was performed at each follow-up visit in participants with no new diagnosis of diabetes, using a 75-g bolus of glucose dissolved in 0.25–0.3 L water. Blood samples were collected after fasting and 2 h after administering the glucose bolus. Glucose (mmol/L) and insulin levels (μU/mL) were measured in each blood sample.

The incidence of type 2 diabetes in the MSC study sample was defined as a new diagnosis during the study follow-up for participants with a fasting plasma glucose ≥7 mmol/L, or as a self-reported new diagnosis made by a health care professional, or starting a new treatment with glucose-lowering drugs between the baseline and follow-up examinations.

#### Covariates

##### Stockholm Diabetes Prevention Program

Age and sex were obtained from the Swedish general population register. The baseline questionnaires included information on family history of type 2 diabetes (defined as at least one first- or two second-degree family members with type 2 diabetes); other chronic comorbidities (dichotomized); general health (reported as very good, good, neither good nor bad, bad, or very bad); physical activity level in comparison to others of the same age (categorized as much lower, lower, average, higher, and much higher); level of education (categorized as primary education, upper-secondary level, and university or higher); and smoking status (categorized as current smoker, previous smoker, or never smoked).

Anthropometric measurements were made by trained study nurses and included height (m), weight (kg), and waist circumference (cm). Body mass index (BMI) was calculated as BMI = weight (kg)/height(m)^2^. Systolic and diastolic blood pressures (mmHg) were measured using an aneroid sphygmomanometer. Insulin resistance (HOMA2-IR) and β-cell function (HOMA2-B) were estimated using fasting glucose and insulin levels, according to the homeostatic model assessment (HOMA2) [21].

Prediabetes was categorized as impaired fasting glucose (IFG), impaired glucose tolerance (IGT), or both, according to the American Diabetes Association (ADA) and WHO definitions. The ADA defines IFG as a fasting glucose of 5.6–6.9 mmol/L, while the WHO defines it as 6.1–6.9 mml/L. Both the ADA and the WHO define IGT as a 2-h glucose of 7.8–11.0 mmol/L.

##### The Metabolic Syndrome Cohort

Self-reported baseline covariates were age, family history of type 2 diabetes (at least one first-degree relative with a diagnosis of type 2 diabetes), physical activity (using the short version of the International Physical Activity Questionnaire, categorized as low, moderate, or vigorous physical activity), level of education (categorized as primary education (<9 years), upper-secondary education (9–12 years), or university and higher (>12 years)), and comorbidities (a previous diagnosis of hypertension, elevated blood cholesterol, or endocrine disease).

Baseline BMI was determined from measured height and weight as above, and systolic and diastolic blood pressures were measured by a trained health worker using an aneroid sphygmomanometer. Fasting blood glucose (mg/dL) and insulin (μU/mL) levels were measured. Fasting glucose was converted to mmol/L according to the formula: *mg/dL × 0.0555 = mmol/L*. Prediabetes was determined based on fasting plasma glucose level and categorized as IFG according to the WHO and ADA criteria, as described above.

### Cluster analysis

Cluster analysis was performed using k-prototypes, an unsupervised partitioning method that divides the dataset based on the variability of categorical and continuous variables [22]. The variables used for the identification of clusters were chosen based on previous studies [14, 16] and included fasting plasma glucose and insulin levels, HOMA2-IR, HOMA2-B, BMI, systolic and diastolic blood pressure, sex, family history of type 2 diabetes, and level of education. Prior to analysis, continuous variables were standardized (mean=0, standard deviation (SD)=1), and extreme outliers, defined as values ≥ ±5 SD, were removed.

Analysis was done independently in the main data (SDPP) and validation data (MSC). We assessed validity based on measures of internal and external validity, internal stability, and visual validation [23]. The gap statistic was used to determine the optimal number of clusters [24] and as a measure of internal validity together with the within clusters sum of squares. External validity was assessed using Cox proportional hazard models to determine whether the clustering process predicts the incidence of type 2 diabetes. The internal stability of the clusters was assessed with the Jaccard similarity index, estimated using 1000 bootstrapped samples. A value greater than 0.75 for each cluster was considered stable [25]. Finally, visual validation was done using box plots and bar charts, as well as uMAP and heatmaps to compare the patterns of the variables used for clustering in each cohort. A detailed description is available as Additional file 3 [22].

### Statistical analysis

The baseline characteristics of the participants, grouped by cluster, are presented as the mean and SD for continuous variables, and as proportions for binary and categorical data (Tables 1 and 2). The risk of type 2 diabetes associated with the resulting clusters was estimated using multivariable Cox proportional hazards models with age as the underlying time variable [26]. The participants in the SDPP study sample were followed from the date of baseline examination to the first recorded date of a new diagnosis of type 2 diabetes, date of death, diagnosis of type 1 diabetes obtained from registers, or until March 31, 2021. The participants in the MSC study sample were followed from the date of baseline examination until the self-reported date of diagnosis of type 2 diabetes, self-reported starting date of glucose-lowering therapy, date of follow-up examination when diagnosed with type 2 diabetes, date of death or until February 28, 2014.

**Table 1:** Baseline characteristics of the clusters derived from SDPP data

	Total *n*=7317	VLR *n*=1891	LRLB *n*=1681	LRHB *n*=1237	HRHBP *n*=731	HRBF *n*=1229	HRIR *n*=548
Mean age, (±SD)	47.10 (4.92)	46.10 (5.20)	48.17 (4.44)	46.01 (5.04)	48.70 (4.09)	47.45 (4.91)	46.76 (4.72)
Women (%)	4442 (60.7%)	1636 (86.5%)	1337 (79.5%)	212 (17.1%)	459 (62.8%)	722 (58.7%)	76 (13.9%)
Men (%)	2875 (39.3%)	255 (13.5%)	344 (20.5%)	1025 (82.9%)	272 (37.2%)	507 (41.3%)	472 (86.1%)
Mean BMI, kg/m^2^ (±SD)	25.55 (3.83)	22.97 (2.45)	24.53 (2.74)	25.33 (2.83)	27.64 (3.95)	27.93 (3.73)	29.99 (4.17)
Overweight (%)	2843 (38.9%)	352 (18.6%)	614 (36.5%)	563 (45.5%)	363 (49.7%)	685 (55.7%)	266 (48.5%)
Obesity (%)	855 (11.7%)	13 (0.7%)	57 (3.4%)	77 (6.2%)	180 (24.6%)	294 (23.9%)	234 (42.7%)
Systolic blood pressure, mmHg (±SD)	122.51 (15.66)	107.78 (7.91)	126.09 (8.85)	120.62 (10.27)	149.72 (13.22)	121.16 (10.13)	133.40 (14.54)
Diastolic blood pressure, mmHg (±SD)	76.89 (9.99)	66.52 (5.16)	79.21 (5.26)	77.47 (7.10)	92.59 (7.81)	75.94 (6.38)	85.48 (8.72)
Fasting glucose, mmol/L (±SD)	4.71 (0.53)	4.52 (0.37)	4.58 (0.35)	4.32 (0.38)	4.88 (0.50)	5.31 (0.42)	5.01 (0.61)
Two-hour glucose, mmol/L (±SD)	4.74 (1.41)	4.24 (1.07)	4.54 (1.16)	4.42 (1.25)	5.33 (1.51)	5.31 (1.50)	5.74 (1.81)
Fasting insulin level, μU/mL (±SD)	14.33 (7.39)	9.41 (3.19)	9.22 (2.72)	19.28 (3.92)	14.46 (4.60)	16.02 (4.06)	31.82 (6.39)
Two-hour insulin level, μU/mL (±SD)	46.68 (32.45)	31.73 (14.77)	33.77 (15.72)	52.75 (29.16)	52.44 (30.69)	53.24 (30.18)	101.82 (52.84)
HOMA2 B (±SD)	147.37 (56.39)	120.82 (31.21)	116.36 (27.83)	216.65 (45.67)	139.95 (37.47)	127.00 (27.20)	233.30 (62.32)
HOMA2 IR (±SD)	1.56 (0.79)	1.03 (0.35)	1.01 (0.30)	2.04 (0.41)	1.59 (0.51)	1.80 (0.45)	3.43 (0.66)
Family history of type 2 diabetes (%)	4278 (58.5%)	1015 (53.7%)	964 (57.3%)	664 (53.7%)	465 (63.6%)	814 (66.2%)	356 (65.0%)
Primary education (%)	2249 (30.7%)	456 (24.1%)	516 (30.7%)	336 (27.2%)	298 (40.8%)	439 (35.7%)	204 (37.2%)
Upper-secondary education (%)	2920 (39.9%)	634 (33.5%)	679 (40.4%)	604 (48.8%)	249 (34.1%)	509 (41.4%)	245 (44.7%)
University or higher (%)	2148 (29.4%)	801 (42.4%)	486 (28.9%)	297 (24.0%)	184 (25.2%)	281 (22.9%)	99 (18.1%)

*SDPP* Stockholm Diabetes Preventive Program, *VLR* very low-risk, *LRLB* low-risk low β-cell function, *LRHB* low-risk high β-cell function, *HRHBP* high-risk high blood pressure, *HRBF* high-risk β-cell failure, *HRIR* high-risk insulin-resistant, *SD* standard deviation

**Table 2:** Baseline characteristics of the clusters derived from MSC data

	Total ***n***=2332	VLR ***n***=559	LRLB ***n***=643	LRHB ***n***=374	HRHBP ***n***=254	HRBF ***n***=360	HRIR ***n***=142
Mean age, (±SD)	42.60 (7.76)	40.66 (7.16)	43.34 (7.70)	40.28 (7.10)	46.65 (7.69)	44.24 (7.93)	41.68 (7.44)
Women (%)	1663 (71.3%)	434 (77.6%)	411 (63.9%)	293 (78.3%)	157 (61.8%)	261 (72.5%)	107 (75.4%)
Men (%)	669 (28.7%)	125 (22.4%)	232 (36.1%)	81 (21.7%)	97 (38.2%)	99 (27.5%)	35 (24.6%)
Mean BMI, kg/m^2^(±SD)	29.15 (4.59)	26.17 (2.91)	27.36 (3.08)	30.88 (4.22)	32.02 (4.62)	31.10 (4.33)	34.39 (5.37)
Overweight (%)	1081 (46.4%)	279 (49.9%)	375 (58.3%)	156 (41.7%)	93 (36.6%)	144 (40.0%)	34 (23.9%)
Obesity (%)	835 (35.8%)	58 (10.4%)	116 (18.0%)	201 (53.7%)	153 (60.2%)	200 (55.6%)	107 (75.4%)
Systolic blood pressure, mmHg (±SD)	114.69 (14.56)	101.17 (8.16)	118.56 (8.12)	113.23 (9.38)	138.84 (13.81)	111.33 (9.60)	119.63 (15.07)
Diastolic blood pressure, mmHg (±SD)	76.51 (10.22)	66.01 (6.19)	80.41 (5.04)	76.74 (7.46)	91.44 (7.50)	74.11 (7.59)	78.98 (11.68)
Fasting glucose, mmol/L (±SD)	4.91 (0.56)	4.61 (0.40)	4.83 (0.40)	4.49 (0.39)	5.17 (0.51)	5.60 (0.39)	5.25 (0.58)
Fasting insulin μU/mL (SD)	12.15 (6.97)	7.94 (2.96)	7.66 (2.53)	15.74 (4.04)	14.00 (4.73)	14.19 (4.18)	31.05 (6.31)
HOMA2 B (±SD)	120.01 (45.97)	103.18 (28.39)	92.16 (23.50)	173.51 (35.57)	121.47 (28.84)	104.57 (22.33)	208.00 (49.66)
HOMA2 IR (±SD)	1.34 (0.77)	0.87 (0.33)	0.85 (0.28)	1.70 (0.44)	1.57 (0.54)	1.62 (0.48)	3.40 (0.68)
Family history of type 2 diabetes (%)	1856 (79.6%)	440 (78.7%)	509 (79.2%)	287 (76.7%)	207 (81.5%)	295 (81.9%)	118 (83.1%)
Primary education (%)	1077 (46.2%)	188 (33.6%)	313 (48.7%)	187 (50.0%)	121 (47.6%)	197 (54.7%)	71 (50.0%)
Upper-secondary education (%)	419 (18.0%)	112 (20.0%)	129 (20.1%)	63 (16.8%)	42 (16.5%)	50 (13.9%)	23 (16.2%)
University or higher (%)	836 (35.8%)	259 (46.3%)	201 (31.3%)	124 (33.2%)	91 (35.8%)	113 (31.4%)	48 (33.8%)

*MSC* Metabolic Syndrome Cohort, *VLR* very low-risk, *LRLB* low-risk low β-cell function, *LRHB* low-risk high β-cell function, *HRHBP* high-risk high blood pressure, *HRBF* high-risk β-cell failure, *HRIR* high-risk insulin resistance, *SD* standard deviation

Proportional hazards were assessed visually using log-log plots of survival and predicted Kaplan–Meier survival plots, as well as statistically. Analysis in SDPP was stratified by year of birth using 5-year intervals [26]. Crude and adjusted hazard ratios (HR) are reported. Possible confounders included in the models were sex, self-reported general health status, presence of chronic comorbidities, physical activity level, smoking status, and history of gestational diabetes among women [27].

To evaluate the clinical utility of the clusters, the predictive accuracy and long-term stability of the clusters were assessed and compared to prediabetes in the SDPP data. Predictive accuracy was evaluated using sensitivity, specificity, area under the curve (AUC), and concordance statistics. The long-term stability of the clusters was assessed in the SDPP data from participants who had attended all the study follow-ups using intra-rater agreement measured as Cohen’s kappa and Gwet’s AC1 index [26].

Statistical analyses were performed using Stata version 17 [28]. The clustering algorithm was implemented using a customized k-prototype package in Python 3.7 [29, 30]. The code used can be found as Additional file 3.

## Results

The participants in the SDPP study sample were followed for an average of 23 years, representing 169031.1 person-years. A total of 1226 incident cases of type 2 diabetes were identified, giving an overall incidence rate of 7.25 (95% CI: 6.86, 7.67) cases per 1000 person-years. The incidence rate in men was 9.01 (95% CI: 8.42, 9.82) cases per 1000 person-years, and in women, 5.95 (95% CI: 5.49, 6.45) cases per 1000 person-years. Self-reporting was the only source of diagnosis for 44 (<5%) individuals. In total, 17 (0.2%) individuals were diagnosed with type 1 or LADA diabetes, and 402 (5.5%) had died during the study.

Participants in the MSC study sample were followed up for a mean of 3.8 years, representing 5679.9 person-years. One hundred thirty-one incident cases of type 2 diabetes were identified. The overall incidence rate was 23.06 (95% CI: 19.43, 27.37) cases per 1000 person-years. The incidence rate was slightly higher for men than for women: 23.48 (95% CI: 17.08, 32.27) vs. 22.89 (95% CI: 18.69, 28.06) cases per 1000 person-years. No deaths were registered during the follow-up period.

**Fig. 1:**
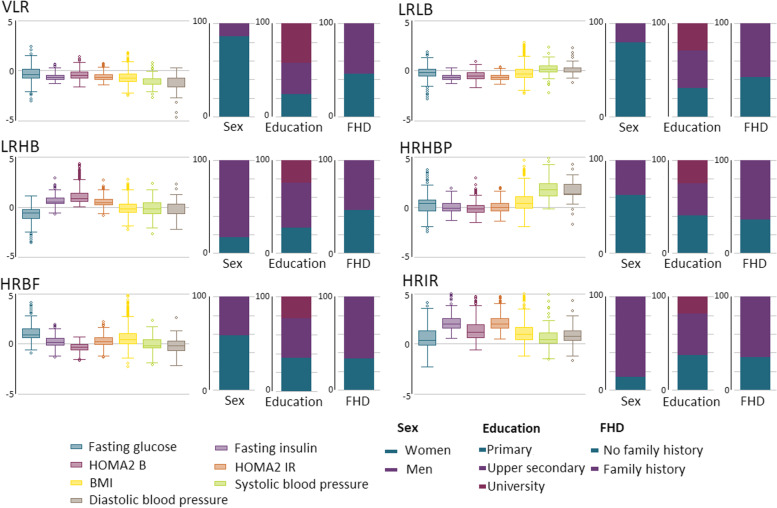
Box plot and bar charts of baseline characteristics among clusters in SDPP. FHD family history of diabetes, VLR very low-risk, LRLB low-risk low β-cell function, LRHB low-risk high β-cell function, HRHBP high-risk high blood pressure, HRBF high-risk with predominance of β-cell failure, HRIR high-risk insulin resistance

**Fig. 2:**
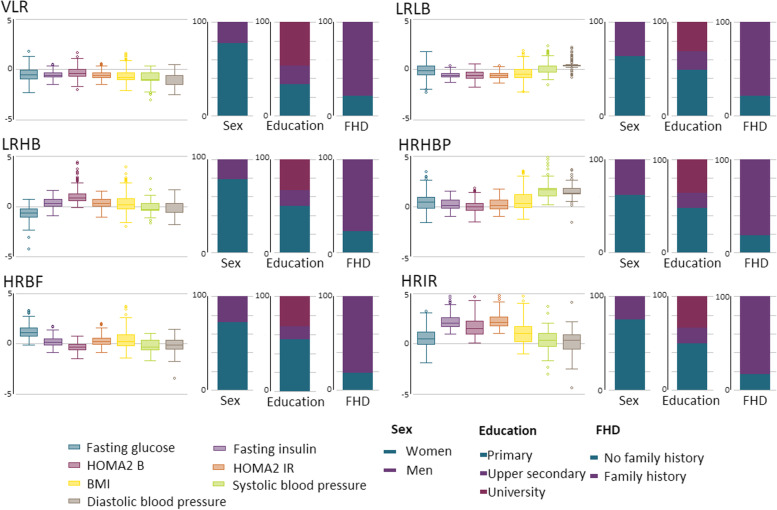
Box plot and bar charts of baseline characteristics among clusters in MSC. FHD family history of diabetes, VLR very low-risk, LRLB low-risk low β-cell function, LRHB low-risk high β-cell function, HRHBP high-risk high blood pressure, HRBF high-risk with predominance of β-cell failure, HRIR high-risk insulin resistance

Cluster analysis of the SDPP and the MSC data resulted in six distinctive clusters, as presented in Figs. 1 and 2. All clusters had good stability (Jaccard similarity index >85% for all clusters in both study samples). Detailed results regarding the determination of the number of clusters, internal validity, and cluster stability are given in Additional file 1: Tables S2-S3, and Additional file 2: Fig. S3. Visually, the two study samples showed similar overall distributions of risk factors in each cluster, leading to comparable phenotypes (see Additional file 2: Figs. S4-S5). We found small yet significant differences in some of the values of the variables used for cluster analysis between the equivalent clusters in SDPP and MSC, reflecting the underlying differences between the two populations (Additional file 2: Fig. S6).

**Fig. 3:**
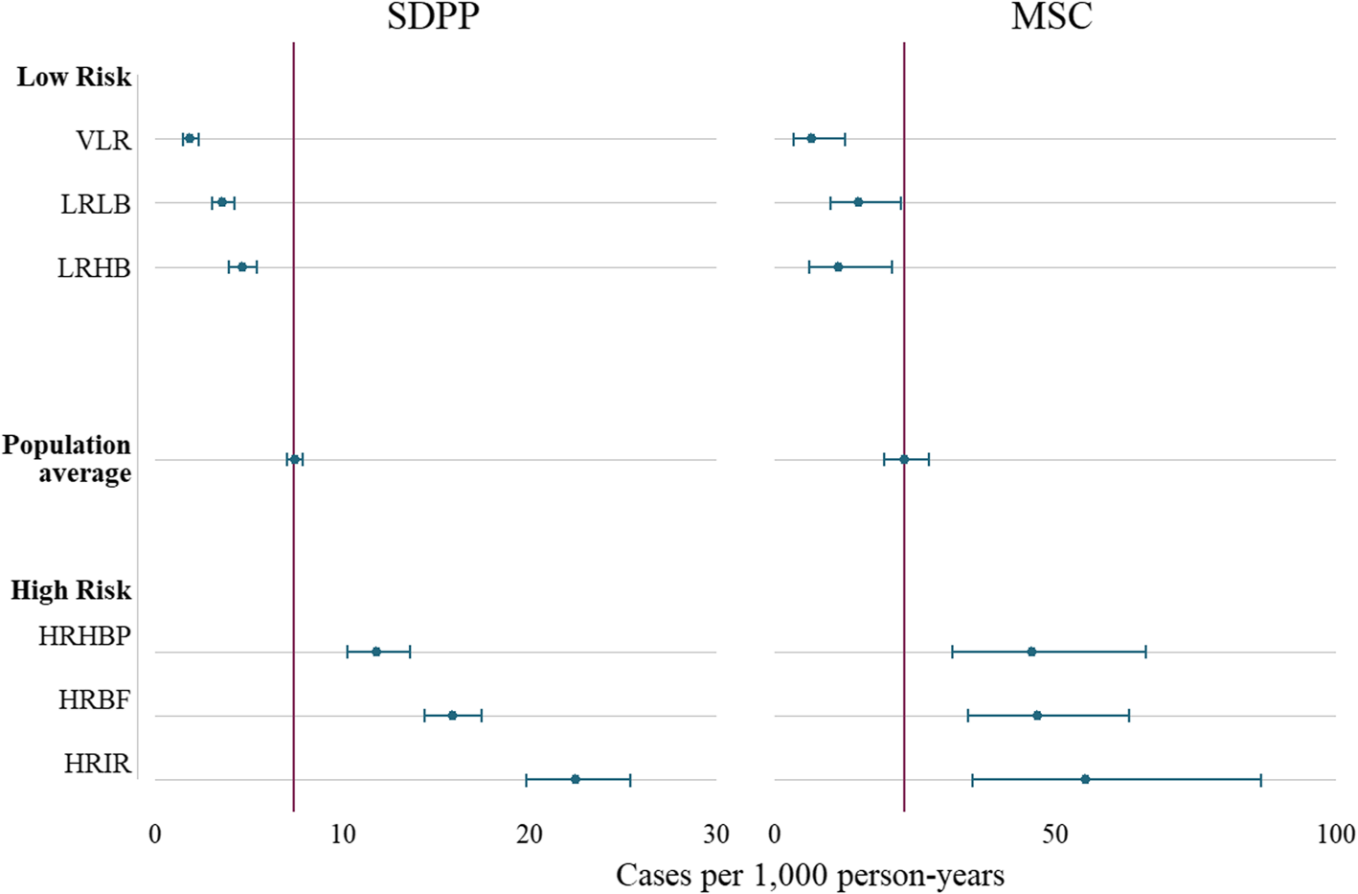
Incidence rates of type 2 diabetes in the SDPP and MSC studies. Compared to the average incidence rate in each study, the clusters divided the populations in three low-risk and three high-risk groups. SDPP Stockholm Diabetes Preventive Program, MSC The Metabolic Syndrome Cohort, VLR very low-risk, LRLB low-risk low beta cell function, LRHB low-risk high beta cell function, HRHBP high-risk high blood pressure, HRBF high-risk beta cell failure, HRIR high-risk insulin resistance

Compared to the average incidence of type 2 diabetes in each study sample, the clusters clearly divided the population into three low-risk clusters: very low-risk (VLR), low-risk low β-cell function (LRLB), low-risk high β-cell function (LRHB), and three high-risk clusters: high-risk high blood pressure (HRHBP), high-risk β-cell failure (HRBF), and high-risk insulin-resistant (HRIR), as shown in Fig. 3.

Of the low-risk clusters, the VLR cluster was characterized by predominantly highly educated, young women, without metabolic risk factors in both the SDPP and MSC study samples. The LRLB cluster included mostly women with a higher age than the population mean, but lower levels of fasting insulin, HOMA2-IR, and HOMA2-B than average in each study sample. The LRHB cluster was characterized by low risk, despite dysregulation of insulin production and sensitivity, and a higher proportion of participants with a high BMI. This cluster included participants with the second-highest values of fasting insulin and HOMA2-B, and a higher proportion of participants with BMI ≥ 35 kg/m^2^, as well as the lowest level of education among the other low-risk clusters in both the SDPP and MSC study samples.

Among the high-risk clusters. The sexes were equally distributed in the HRHBP cluster and included participants with the highest mean age and the highest levels of systolic and diastolic blood pressure, in both study samples. In the HRBF cluster, there was a predominance of women, the highest fasting glucose levels, and second lowest HOMA2-B, together with the highest proportion of individuals with a family history of type 2 diabetes, in both study samples. Finally, the HRIR cluster consisted mostly of men in the SDPP and women in the MSC study sample, who had the second-highest proportion of family history of diabetes and the highest values of HOMA2-B and HOMA2-IR.

**Fig. 4:**
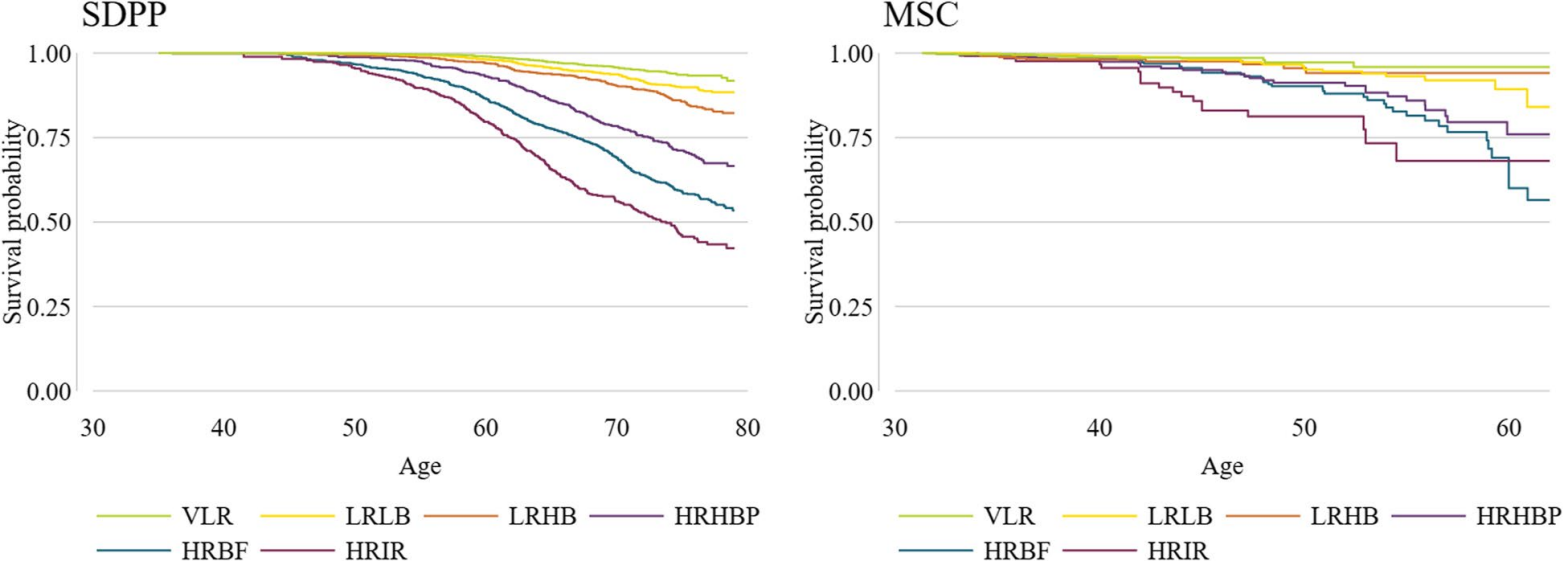
Kaplan–Meier estimates of the risk of type 2 diabetes. SDPP Stockholm Diabetes Prevention Program, MSC Metabolic Syndrom Cohort, VLR very low-risk, LRLB low-risk low beta cell function, LRHB low-risk high beta cell function, HRHBP high-risk high blood pressure, HRBF high-risk beta cell failure, HRIR high-risk insulin resistance

Survival analysis showed similar trends of the association between cluster membership and incidence of type 2 diabetes in both cohorts. Detailed results are presented in Table 3 and Fig. 4. In the SDPP sample, compared to the LRHB cluster, a statistically significant inverse association was found between the VLR (HR: 0.38 95% CI: 0.28, 0.50) and the LRLB (HR: 0.71, 95% CI: 0.55, 0.90) clusters and incidence of type 2 diabetes. While a statistically significant positive association was found between the HRHBP (HR: 2.34, 95 CI: 1.85, 2.96), the HRBF (HR: 3.22, 95% CI: 2.62, 3.96), and the HRIR (HR: 5.39, 95% CI: 4.30, 6.75) clusters and incidence of type 2 diabetes.

**Table 3:** Results of the Cox proportional hazards models

	SDPP					MSC				
	Person-time	Cases	Rate per 1000 (95% CI)	Unadjusted HR (95% CI)	Adjusted HR (95% CI)	Person-time	Cases	Rate per 1000 (95% CI)	Unadjusted HR (95% CI)	Adjusted HR (95% CI)
**VLR**	44654.0	82	1.84 (1.48, 2.28)	0.41^***^ (0.32, 0.54)	0.38^***^ (0.28, 0.50)	1399.40	9	6.43 (3.35, 12.36)	0.55^***^ (0.51, 0.60)	0.58^***^ (0.51, 0.66)
**LRLB**	39519.7	141	3.57 (3.02, 4.21)	0.75^*^ (0.59, 0.95)	0.71^**^ (0.55, 0.90)	1542.28	23	14.91 (9.91, 22.44)	1.23 (0.51, 2.97)	1.24 (0.50, 3.12)
**LRHB**	31220.8	144	4.61 (3.91, 5.43)	Ref (1.00)	Ref (1.00)	891.15	10	11.22 (6.04, 20.86)	Ref (1.00)	Ref (1.00)
**HRHBP**	16167.2	191	11.81 (10.25, 13.61)	2.50^***^ (2.01, 3.11)	2.34^***^ (1.85, 2.96)	620.84	28	45.10 (31.14, 65.32)	3.39^**^ (1.55, 7.41)	3.26^**^ (1.49, 7.15)
**HRBF**	26333.1	418	15.87 (14.42, 17.47)	3.58^***^ (2.96, 4.33)	3.22^***^ (2.62, 3.96)	885.16	42	47.45 (35.07, 64.21)	3.96^***^ (2.04, 7.68)	4.00^***^ (2.05, 7.82)
**HRIR**	11136.3	250	22.45 (19.83, 25.41)	5.31^***^ (4.32, 6.52)	5.39^***^ (4.30, 6.75)	343.10	19	55.38 (35.32, 86.82)	4.74^**^ (1.70, 13.22)	4.52^***^ (1.66, 12.32)
**Total**	169031.1	1226	7.25 (6.86, 7.67)			5681.93	131	23.06 (19.43, 27.37)		

*SDPP* Stockholm Diabetes Prevention Program, *MSC* Metabolic Syndrome Cohort, *HR* hazard ratio, *95% CI* 95% confidence interval, *VLR* very low-risk, *LRLB* low-risk low β-cell function, *LRHB* low-risk high β-cell function, *HRHBP* high-risk high blood pressure, *HRBF* high-risk with predominance of β-cell failure, *HRIR* high-risk insulin resistance, *p < 0.05, **p < 0.01, ***p < 0.001. Model 1: unadjusted. Model 2: adjusted for self-reported general health (in SDPP), comorbidity, self-reported physical activity level, smoking status, and history of gestational diabetes among women

In the MSC study sample, a statistically significant inverse association was found between the VLR cluster (HR: 0.58, 95% CI: 0.51, 0.66) and the incidence of type 2 diabetes. No association was found between the LRLB cluster (HR: 1.24, 95% CI: 0.50, 3.12), and a significantly positive association was found between the HRHBP (HR: 3.26, 95% CI: 1.49, 7.15), the HRBF (HR: 4.00, 95% CI: 2.05, 7.82), and the HRIR (HR: 4.52, 95% CI: 1.66, 12.32) clusters and incidence of type 2 diabetes. Results of the pairwise comparisons can be found in Additional file 1: Table S4.

In data from SDPP, of the 2508 (34.3%) participants categorized in a high-risk cluster at baseline, 859 (34.3%) progressed to type 2 diabetes during the study follow-up. While of the 650 (8.9%) participants with ADA-defined prediabetes at baseline, 239 (56%) progressed to type 2 diabetes. And of 374 (5.1%) with WHO defined, 239 (63.9%) progressed to type 2 diabetes (see also Additional file 2: Fig. S7).

**Fig. 5:**
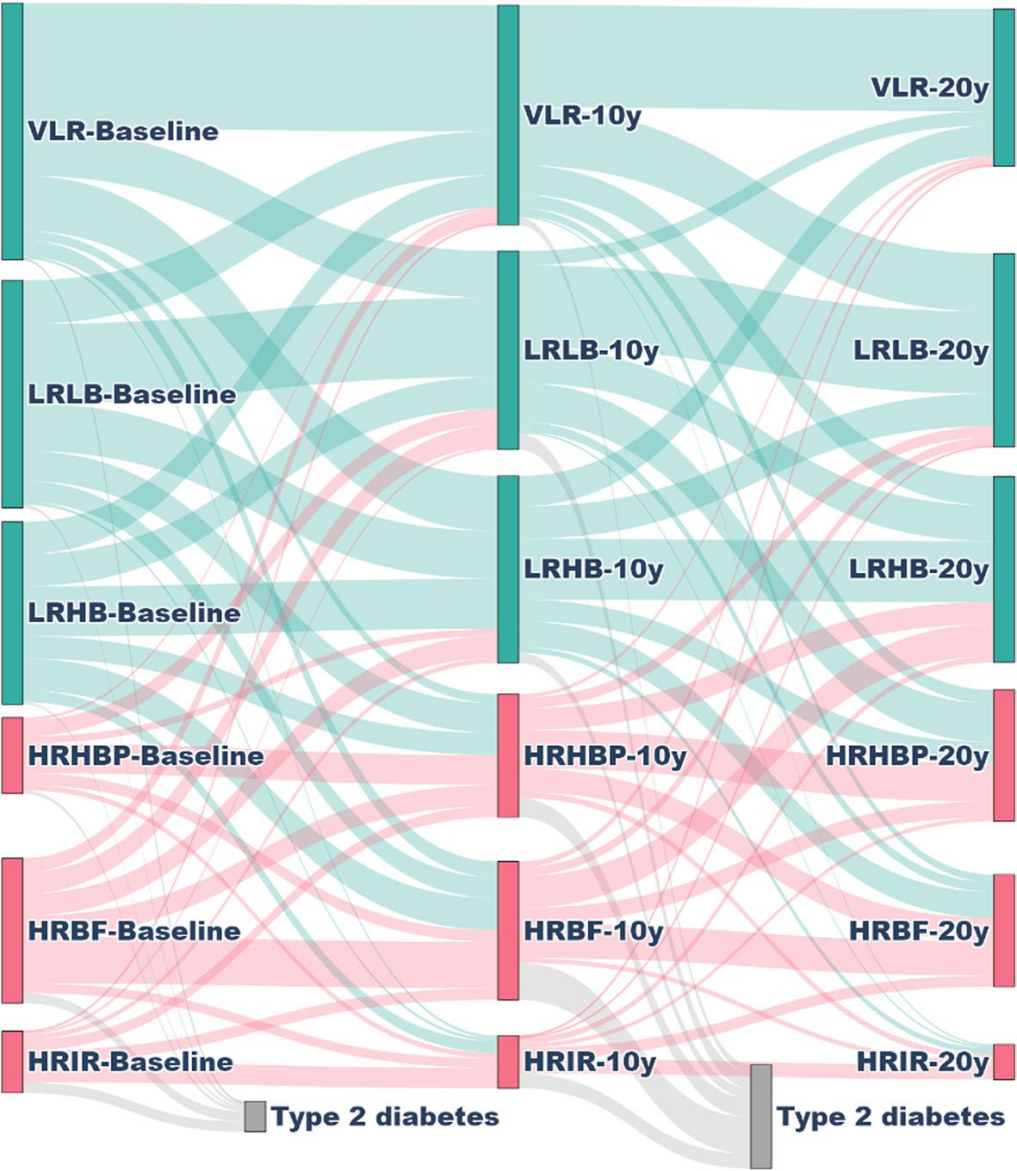
Transition plot of the clinical clusters in the SDPP cohort. Patterns of transition between the baseline, 10-year, and 20-year follow-ups. The thickness of the line represents the proportion of individuals at each time point. Low-risk clusters are marked in light blue while the high-risk clusters in red. VLR very low-risk, LRLB low-risk low beta cell function, LRHB low-risk high beta cell function, HRHBP high-risk high blood pressure, HRBF high-risk beta cell failure, HRIR high-risk insulin resistance

Complete data from all follow-ups was available from a subsample of 3379 (46.1%) participants. Of 1033 (30.6%) participants in a high-risk cluster at baseline, 407 (39.4%) remained in a high-risk cluster, 280 (27.1%) regressed to a low-risk cluster, and 346 (33.4%) progressed to type 2 diabetes. While of 226 (6.7%) with ADA-defined prediabetes at baseline, 65 (28.8%) remained stable, 28 (12.4%) regressed to normal glycemia, and 133 (58.9%) progressed to type 2 diabetes. And from 124 (3.7%) with WHO prediabetes at baseline, 17 (13.7%) remained stable, 21 (16.9%) regressed to normal glycemia, and 86 (69.4%) were diagnosed with type 2 diabetes. Transitions from the different clusters to type 2 diabetes are presented in Fig. 5 and from prediabetes to type 2 diabetes in Additional file 2: Fig. S8.

The predictive accuracy of the high-risk clusters as a group was high compared to that of both definitions of prediabetes, with an AUC of 0.71 (95% CI: 0.70, 0.73) for the high-risk clusters, 0.63 (95% CI: 0.61, 0.64) for the ADA definition, and 0.59 (95% CI: 0.58, 0.60) for the WHO definition of prediabetes. Sensitivity (70.1%, 95% CI: 67.4%, 72.6%) and specificity (72.9%, 95% CI: 71.8%, 74.0%) were both high for the high-risk clusters. In contrast, prediabetes showed a low sensitivity: 29.9% (95% CI: 27.4%, 32.6%) for the ADA and 19.5% (95% CI: 17.3%, 21.8%) for the WHO definitions, and high specificity ranging from 95.3% (95% CI: 94.8%, 95.9%) for the ADA to 97.8% (95% CI: 97.4%, 98.2%) for the WHO definitions, as summarized in Additional file 1: Tables S5-S7.

In general, agreement of high-risk clusters ranged from fair to moderate, while both the ADA and WHO definitions of prediabetes showed a fair to high agreement (see Additional file 1: Table S8).

## Discussion

We explored the utility of cluster analysis based on common risk factors for type 2 diabetes. Using data from two independent longitudinal studies, we found six characteristic clusters (three low-risk and three high-risk) that were useful to stratify the risk of type 2 diabetes in both cohorts. Compared to different definitions of prediabetes, the high-risk clusters had a better predictive accuracy and were stable after over 20 years of follow-up.

### Comparison with previous studies

Previous studies have used similar methods to investigate the heterogeneity of type 2 diabetes and its complications. Li and collaborators reported three distinct phenotypes of type 2 diabetes using data from digital medical records [13], and Ahlqvist et al. more recently described five clusters of type 2 diabetes using cluster analysis [14]. However, while the clusters reported by Ahlqvist et al. have been replicated in some studies [31, 32], others have failed to reproduce them or found their clinical utility to be limited [33, 34].

Few studies describe clusters before the diagnosis of type 2 diabetes. A study in South Korea by Cho et al. identified six different clusters associated with differences in the prevalence of type 2 diabetes [17], and another by Wagner and collaborators applied cluster analysis to detect phenotypes among individuals at high risk for type 2 diabetes [35].

In all the studies mentioned above, apart from that by Cho et al., analysis required complex data (such as the presence of antibodies to glutamic acid decarboxylase) which are not readily available in most settings. While such data are very valuable, if the aim is to implement cluster analysis widely for the surveillance and prevention of type 2 diabetes, data should be accessible.

On the other hand, important risk factors such as sex and socioeconomic position have not consistently been included, perhaps due to the methodological challenge of using categorical values in cluster analysis. Only the study by Cho et al. included sex, while no previous studies have used indicators of socioeconomic position. In our study, female sex and higher education were overrepresented in the low-risk clusters, indicating the importance of sex and socioeconomic status in the pathophysiological process leading to type 2 diabetes.

The degree of replicability in previous studies is unclear. In the study by Wagner et al., the clusters were replicated using a larger cohort, although they used different variables, assuming that they were conceptually similar, while Ahlqvist et al. and Cho et al. used replication samples derived from the same target population. In contrast, we used comparable data from two independent populations and found comparable patterns.

Additionally, it remains unclear whether different clusters represent different stages in the natural history of type 2 diabetes or etiologically different phenotypes. Although studies have reported the short-term transitions between clusters through time [35, 36], our study is, to the best of our knowledge, the first describing long-term stability.

Our clusters also show important similarities to those reported previously. For example, the phenotypes defined by Ahlqvist et al., severe insulin-deficient diabetes and severe insulin-resistant diabetes, and by Wagner et al. with a prominence of β-cell failure and insulin resistance, resemble our HRBF and HRIR clusters. The studies by Cho et al. and Li et al. describe a subgroup characterized by high blood pressure, like the HRHBP cluster described in this study. The mild obesity-related diabetes cluster described by Ahlqvist et al. and the low-risk obese subgroup described by Wegner et al. are similar to the LRHB cluster, which includes individuals with higher BMI than the population average who had a relatively low risk of type 2 diabetes. However, this was not the most notable characteristic of this group in our study. This difference might be explained by selection bias introduced by clustering individuals with type 2 diabetes or at high risk of type 2 diabetes, among whom high BMI is overrepresented.

### Strengths and limitations

The strengths of this study include the use of independent study samples from different countries, which allowed to assess the replicability of our findings. Furthermore, a large and representative sample with a follow-up period of over 20 years (SDPP) was used for the main analysis, which allowed us to estimate the incidence of type 2 diabetes with limited attrition, thanks to the availability of data from regional and national registers.

The replication sample (MSC), in contrast, had a shorter follow-up (3 to 6 years), had a relatively low number of new cases of type 2 diabetes, and was more prone to bias due to sample selection and attrition, which might impact external validation, as the statistical power of the survival analysis to detect an association between cluster assignment and risk of type 2 diabetes might be limited. However, the data demonstrated trends consistent with those of SDPP. Replication using data with longer follow-ups is needed to ensure the external validity of our findings. Likewise, the secondary analysis of the long-term stability on a subset from SDPP might also be biased by loss to follow-up. These results should thus be interpreted with caution.

The selection of risk factors used in the cluster analysis was based in previous literature and included well-known risk factors of type 2 diabetes. Data on biochemical and genetics were not available, and it is unclear whether it could have affected the results of the cluster analysis. Nevertheless, such information is not usually available in most settings.

Incorrect classification of type 2 diabetes as type 1 or other sorts of diabetes is a common problem in epidemiological studies [37]. In SDPP, data from clinical registries allowed us to identify individuals with other types of diabetes directly. In the MSC study, we limited the sample to individuals with a diagnosis after 30 years of age to minimize the risk of including individuals with type 1 diabetes. However, neither method completely eliminates the risk of misclassification bias.

### Implications for future research and public health

From a public health perspective, data-driven risk stratification could lead to better-targeted interventions and resource utilization for the prevention of type 2 diabetes. The high-risk clusters described in this study define a group with high sensitivity and specificity that contains about one-third of the study population and captures a large majority of the cases of type 2 diabetes, which might have important implications for public health and clinical practice. However, questions remain regarding the practicality of using cluster analysis, its utility for guiding public health policy and clinical decisions, and the underlying physiological mechanisms driving the different phenotypes.

The first step towards the practical implementation of data-driven stratification is assigning individuals to the most appropriate cluster. This can be accomplished using data available in health registers or electronic medical records. The observed differences between the Swedish and Mexican cohorts indicate that clustering should be population specific, although our results show that this is likely to result in very similar phenotypes.

Clusters could also be used to investigate the effect of different interventions to prevent type 2 diabetes. For example, the relatively high blood pressure in the HRHBP cluster suggests that blood pressure control could be an important part of the intervention for this group of individuals. The high fasting glucose and insulin levels of individuals in the HRBF cluster resemble the β-cell dysfunction characteristic of type 2 diabetes; thus, pharmacological interventions to regulate β-cell function might be effective. On the other hand, intensive lifestyle interventions might have the greatest effects in individuals in the HRIR cluster, who were characterized by the highest BMI and tendencies towards insulin resistance.

Likewise, studies looking into the association between different clusters and complications of type 2 diabetes may aid clinical decision-making in patients with type 2 diabetes and lead to new insights into the natural history and pathophysiology of the disease.

Finally, to better understand the physiological mechanisms underlying the differences between clusters, studies of the environmental, social, genetic, and biochemical determinants of the different clusters are needed.

## Conclusions

Phenotypes derived using cluster analysis on readily available risk factors in two independent longitudinal studies from Sweden and Mexico were useful to stratify the risk of type 2 diabetes among diabetes-free adults. The validity and reliability of the clusters described in this study, compared to those of prediabetes, indicate their potential clinical utility. These results could be used to develop more precise interventions to prevent type 2 diabetes.

## Supplementary Information

**Additional file 1: Table S1.** Baseline characteristics of the SDPP and MSC studies. **Table S2.** Determination of the number of clusters in the SDPP and MCS studies. **Table S3.** Mean Jaccard similarity of the cluster categories in the SDPP and MSC studies. **Table S4.** Pairwise comparisons of the association between cluster membership and incidence of type 2 diabetes. **Table S5.** Accuracy of categories of prediabetes to predict type 2 diabetes in the SDPP study. **Table S6.** Accuracy of individual clusters to predict type 2 diabetes in the SDPP study. **Table S7.** Accuracy of prediabetes and high-risk clusters to predict type 2 diabetes in the SDPP study. **Table S8.** Intrarater reliability of clusters and prediabetes in the SDPP study.

**Additional file 2: Figure S1.** Flow chart of the Stockholm Diabetes Preventive Program (SDPP). **Figure S2.** Flow chart of The Metabolic Syndrome Cohort (MSC). **Figure S3.** Determination of the number of clusters using Gap Statistic and within clusters sum of squares. **Figure S4.** Visualization of the clusters in SDPP (panel a) and MSC (panel b) using dimension reduction with u-MAP. **Figure S5.** Heatmap of the distribution of continuous variables by cohort. **Figure S6.** Box plots of the continuous variables used for cluster analysis, comparison between the SDPP and MSC cohorts. **Figure S7.** Baseline categorization as prediabetes and high-risk clusters and cases of type 2 diabetes in the SDPP study. **Figure S8.** Patterns of transition of prediabetes between the baseline, 10-year and 20-year follow-ups of the SDPP cohort.

**Additional file 3.** Detailed description of methods for cluster analysis and computer code (Stata and Python).

## Data Availability

The code used for data analysis is available online as Additional file 3. Data used for this study are available upon reasonable request. Data from SDPP is protected by the General Data Protection Regulation of the European Union. To obtain deidentified data for research purposes, interested researchers can contact the Center for Epidemiology and Community Medicine in Stockholm for SDPP data (ces.slso@regionstockholm.se) and the Intituto Nacional de Ciencias Medicas y Nutrición for data from the Metabolic Syndrome Cohort (carlos.aguilars@incmnsz.mx).

## Abbreviations

ADA: American Diabetes Association
AUC: Area under the curve
BMI: Body mass index
CES: Center for Epidemiology and Community Medicine
CI: Confidence interval
HR: Hazard ratio
HRBF: High-risk β-cell failure
HRHBP: High-risk high blood pressure
HRIR: High-risk insulin-resistant
IFG: Impaired fasting glucose
IGT: Impaired glucose tolerance
LADA: Latent autoimmune diabetes in adults
LRHB: Low-risk high β-cell function
LRLB: Low-risk low β-cell function
MSC: Metabolic Syndrome Cohort
NDR: National Diabetes Registry of Sweden
OGTT: Oral glucose tolerance tests
SD: Standard deviation
SDPP: Stockholm Diabetes Prevention Program
VAL: Clinical inpatient and outpatient registers of Region Stockholm
VLR: Very low-risk
WHO: World Health Organization

## References

[1] James SL, Abate D, Abate KH, Abay SM, Abbafati C, Abbasi N, Abbastabar H, Abd-Allah F, Abdela J, Abdelalim A (2018). Global, regional, and national incidence, prevalence, and years lived with disability for 354 diseases and injuries for 195 countries and territories, 1990-2017: a systematic analysis for the Global Burden of Disease Study 2017. Lancet.

[2] Beagley J, Guariguata L, Weil C, Motala AA (2014). Global estimates of undiagnosed diabetes in adults. Diabetes Res Clin Pract.

[3] Magliano DJ, Islam RM, Barr ELM, Gregg EW, Pavkov ME, Harding JL, Tabesh M, Koye DN, Shaw JE (2019). Trends in incidence of total or type 2 diabetes: systematic review. BMJ.

[4] Eckel RH, Kahn SE, Ferrannini E, Goldfine AB, Nathan DM, Schwartz MW, Smith RJ, Smith SR (2011). Obesity and type 2 diabetes: what can be unified and what needs to be individualized?. Diabetes Care.

[5] Tabak AG, Herder C, Rathmann W, Brunner EJ, Kivimaki M (2012). Prediabetes: a high-risk state for diabetes development. Lancet.

[6] Richter B, Hemmingsen B, Metzendorf MI, Takwoingi Y (2018). Development of type 2 diabetes mellitus in people with intermediate hyperglycaemia. Cochrane Database Syst Rev.

[7] Kivimäki M, Tabák AG (2018). Does addressing prediabetes help to improve population health?. Lancet Diabetes Endocrinol.

[8] World Health Organization: Classification of diabetes mellitus. 2019.

[9] Fitipaldi H, McCarthy MI, Florez JC, Franks PW (2018). A global overview of precision medicine in type 2 diabetes. Diabetes.

[10] Prasad RB, Groop L (2019). Precision medicine in type 2 diabetes. J Intern Med.

[11] Khoury MJ, Iademarco MF, Riley WT (2016). Precision public health for the era of precision medicine. Am J Prev Med.

[12] Bonnefond A, Froguel P (2021). Clustering for a better prediction of type 2 diabetes mellitus. Nat Rev Endocrinol.

[13] Li L, Cheng W-Y, Glicksberg BS, Gottesman O, Tamler R, Chen R, Bottinger EP, Dudley JT (2015). Identification of type 2 diabetes subgroups through topological analysis of patient similarity. Sci Transl Med.

[14] Ahlqvist E, Storm P, Karajamaki A, Martinell M, Dorkhan M, Carlsson A, Vikman P, Prasad RB, Aly DM, Almgren P (2018). Novel subgroups of adult-onset diabetes and their association with outcomes: a data-driven cluster analysis of six variables. Lancet Diabetes Endocrinol.

[15] Safai N, Ali A, Rossing P, Ridderstrale M (2018). Stratification of type 2 diabetes based on routine clinical markers. Diabetes Res Clin Pract.

[16] Harding A-H, Griffin SJ, Wareham NJ (2006). Population impact of strategies for identifying groups at high risk of type 2 diabetes. Prev Med.

[17] Cho SB, Kim SC, Chung MG (2019). Identification of novel population clusters with different susceptibilities to type 2 diabetes and their impact on the prediction of diabetes. Sci Rep.

[18] Gudjonsdottir H, Tynelius P, Fors S, Yacamán Méndez D, Gebreslassie M, Zhou M, et al. Cohort profile: the Stockholm Diabetes Prevention Programme (SDPP). Int J Epidemiol. 2022. 10.1093/ije/dyac147 PMC9749716 35820020

[19] Arellano-Campos O, Gómez-Velasco DV, Bello-Chavolla OY, Cruz-Bautista I, Melgarejo-Hernandez MA, Muñoz-Hernandez L, Guillén LE, Garduño-Garcia JJ, Alvirde U, Ono-Yoshikawa Y (2019). Development and validation of a predictive model for incident type 2 diabetes in middle-aged Mexican adults: the metabolic syndrome cohort. BMC Endocr Disord.

[20] American Diabetes Association (2020). Classification and diagnosis of diabetes: standards of medical care in diabetes 2020. Diabetes Care.

[21] Wallace TM, Levy JC, Matthews DR (2004). Use and abuse of HOMA modeling. Diabetes Care.

[22] Huang Z (1998). Extensions to the k-means algorithm for clustering large data sets with categorical values. Data Min Knowl Disc.

[23] Ullmann T, Hennig C, Boulesteix A-L (2022). Validation of cluster analysis results on validation data: a systematic framework. WIREs Data Mining and Knowledge Discovery.

[24] Tibshirani R, Walther G, Hastie T (2001). Estimating the number of clusters in a data set via the gap statistic. J R Stat Soc Series B (Statistical Methodology).

[25] Hennig C (2007). Cluster-wise assessment of cluster stability. Computational Stat Data Anal.

[26] Korn EL, Graubard BI, Midthune D (1997). Time-to-event analysis of longitudinal follow-up of a survey: choice of the time-scale. Am J Epidemiol.

[27] Laakso M (2019). Biomarkers for type 2 diabetes. Mol Metab.

[28] Statacorp: Stata Statistical Software: release 15. In. College Station, TX: StataCorp LLC; 2017.

[29] KPrototypes_plus [https://github.com/youbao88/KPrototypes_plus].

[30] McInnes L, Healy J, Saul N, Grossberger L. UMAP: uniform manifold approximation and projection. J Open Source Software. 3(29):861.

[31] Kahkoska AR, Geybels MS, Klein KR, Kreiner FF, Marx N, Nauck MA, Pratley RE, Wolthers BO, Buse JB (2020). Validation of distinct type 2 diabetes clusters and their association with diabetes complications in the DEVOTE, LEADER and SUSTAIN-6 cardiovascular outcomes trials. Diabetes Obes Metab.

[32] Bello-Chavolla OY, Bahena-López JP, Vargas-Vázquez A, Antonio-Villa NE, Márquez-Salinas A, Fermín-Martínez CA, Rojas R, Mehta R, Cruz-Bautista I, Hernández-Jiménez S (2020). Clinical characterization of data-driven diabetes subgroups in Mexicans using a reproducible machine learning approach. BMJ Open Diabetes Res Care.

[33] Dennis JM, Shields BM, Henley WE, Jones AG, Hattersley AT (2019). Disease progression and treatment response in data-driven subgroups of type 2 diabetes compared with models based on simple clinical features: an analysis using clinical trial data. Lancet Diabetes Endocrinol.

[34] Lugner M, Gudbjörnsdottir S, Sattar N, Svensson AM, Miftaraj M, Eeg-Olofsson K, Eliasson B, Franzén S (2021). Comparison between data-driven clusters and models based on clinical features to predict outcomes in type 2 diabetes: nationwide observational study. Diabetologia.

[35] Wagner R, Heni M, Tabák AG, Machann J, Schick F, Randrianarisoa E, et al. Pathophysiology-based subphenotyping of individuals at elevated risk for type 2 diabetes. Nat Med. 2021. 10.1038/s41591-020-1116-9 33398163

[36] Zaharia OP, Strassburger K, Strom A, Bönhof GJ, Karusheva Y, Antoniou S, Bódis K, Markgraf DF, Burkart V, Müssig K (2019). Risk of diabetes-associated diseases in subgroups of patients with recent-onset diabetes: a 5-year follow-up study. Lancet Diabetes Endocrinol.

[37] Shields BM, Peters JL, Cooper C, Lowe J, Knight BA, Powell RJ, Jones A, Hyde CJ, Hattersley AT (2015). Can clinical features be used to differentiate type 1 from type 2 diabetes? A systematic review of the literature. BMJ Open.

[38] World Medical Association (2013). World Medical Association Declaration of Helsinki: ethical principles for medical research involving human subjectsWorld Medical Association Declaration of HelsinkiSpecial Communication. JAMA.

