# Supplementary material for: Characterization of data-driven clusters in diabetes-free adults and their utility for risk stratification of type 2 diabetes
Source: BMC Med. 2022 Oct 18;20:356. doi: 10.1186/s12916-022-02551-6 (PMC9578256; doi:10.1186/s12916-022-02551-6)
Supplement: Supplementary file 1 — Additional file 1: Table S1. Baseline characteristics of the SDPP and MSC studies. Table S2. Determination of the number of clusters in the SDPP and MCS studies. Table S3. Mean Jaccard similarity of the cluster categories in the SDPP and MSC studies. Table S4. Pairwise comparisons of the association between cluster membership and incidence of type 2 diabetes. Table S5. Accuracy of categories of prediabetes to predict type 2 diabetes in the SDPP study. Table S6. Accuracy of individual clusters to predict type 2 diabetes in the SDPP study. Table S7. Accuracy of prediabetes and high-risk clusters to predict type 2 diabetes in the SDPP study. Table S8. Intrarater reliability of clusters and prediabetes in the SDPP study. [file 12916_2022_2551_MOESM1_ESM.docx]

Characterization of data-driven clusters in diabetes-free adults and their utility for risk stratification of type 2 diabetes

Diego Yacaman Mendez^a,b^, Minhao Zhou^b^, Ylva Trolle Lagerros^c,d^, Donaji V. Gómez Velasco^e^, Per Tynelius^a,b^, Hrafnhildur Gudjonsdottir^a,b^, Antonio Ponce de Leon^b^, Katarina Eeg-Olofsson^f^ Claes-Göran Östenson, Boel Brynedal^a,b^, Carlos A. Aguilar Salinas^e^, David Ebbevi^a,b^, Anton Lager^a,b^

^a^ Department of Global Public Health, Karolinska Institutet. Stockholm, Sweden.

^b^ Centre for Epidemiology and Community Medicine (CES), Stockholm Health Care Services, Stockholm, Sweden.

^c^ Obesity Centre, Academic Specialist Centre, Stockholm Health Care Services, Stockholm, Sweden.

^d^ Unit of Clinical Epidemiology, Department of Medicine, Karolinska Institutet, Stockholm, Sweden.

e Unidad de Investigación de Enfermedades Metabólicas, Instituto Nacional de Ciencias Médicas y Nutrición “Salvador Zubirán”, Mexico City, Mexico.

f Department of Medicine, Sahlgrenska Academy, University of Gothenburg, Gothenburg, Sweden.

Correspondence to: Diego Yacaman-Mendez MD, MSc.

Department of Global Public Health, Karolinska Institutet, Stockholm, Sweden. SE-171 77.

Email address: diego.yacaman.mendez@ki.se

Declarations of interest: none.

**ADDITIONAL FILE 1: Supplementary tables**

**Table S1.** Baseline characteristics of the SDPP and MSC studies.

|  | **SDPP ( n=7,317)** | **MSC (n=2,332)** |
| --- | --- | --- |
| **Mean age (SD)** | 47.10 (4.92) | 42.60 (7.76) |
| **Women** | 4,442 (60.7%) | 1,663 (71.3%) |
| **Men** | 2,875 (39.3%) | 669 (28.7%) |
| **Mean BMI (kg/m^2^)** | 25.55 (3.83) | 29.15 (4.59) |
| **BMI categories (%)** |  |  |
| **Normal** | 3,619 (49.5%) | 416 (17.8%) |
| **Overweight** | 2,843 (38.9%) | 1,081 (46.4%) |
| **Obesity** | 855 (11.7%) | 835 (35.8%) |
| **History of gestational diabetes (%)** | 169 (2.3%) | 25 (1.1%) |
| **Systolic blood pressure mmHg (SD)** | 122.51 (15.66) | 114.69 (14.56) |
| **Diastolic blood pressure mmHg (SD)** | 76.89 (9.99) | 76.51 (10.22) |
| **Fasting glucose mmol/L (SD)** | 4.71 (0.53) | 4.91 (0.56) |
| **Two-hour glucose mmol/L (SD)** | 4.74 (1.41) | NA |
| **Fasting insulin µU/ml (SD)** | 14.33 (7.39) | 12.15 (6.97) |
| **Two-hour insulin µU/ml (SD)** | 46.68 (32.45) | NA |
| **HOMA2-B** | 147.37 (56.39) | 120.01 (45.97) |
| **HOMA2-IR** | 1.56 (0.79) | 1.34 (0.77) |
| **Family history of type 2 diabetes (%)** | 4,278 (58.5%) | 1,856 (79.6%) |
| **Self-reported comorbidities (%)** | 1,846 (27.8%) | 1,019 (43.7%) |
| **Level of education (%)** |  |  |
| **Primary education** | 2,249 (30.7%) | 1,077 (46.2%) |
| **Upper secondary level** | 2,920 (39.9%) | 419 (18.0%) |
| **University or higher** | 2,148 (29.4%) | 836 (35.8%) |
| **Self-reported physical activity (%)^*^** |  |  |
| **Much lower/ Low** | 791 (10.8%) | 1,285 (55.1%) |
| **Somewhat lower / Moderate** | 3,989 (54.6%) | 579 (24.8%) |
| **Similar/ Vigorous** | 1,957 (26.8%) | 468 (20.1%) |
| **Somewhat or much more** | 575 ( 7.9%) |  |
| **Current smoking (%)** | 1,925 (26.3%) | 566 (29.9%) |
| **Prediabetes (ADA/ WHO)** | 654 (8.9%) / 374(5.1%) | NA |
| **IFG** | 374 (5.1%)/ 94 (1.3%) | 322 (13.8%)/ 70 (3.0%) |
| **IGT** | 191 (2.6%)/ 248 (3.4%) | NA |
| **IFG+IGT** | 89 (1.2%)/ 32(0.4%) | NA |

SDPP: Stockholm Diabetes Preventive Program, MSC: Metabolic Syndrome Cohort, SD: Standard deviation, ADA: American Diabetes Association, IFG: Impaired fasting glucose, IGT: Impaired glucose tolerance . * Self-reported physical activity in SDPP is categorized as “Much lower, Somewhat lower, Similar and Somewhat/much more physical activity” based on self reported questionnaires, while in MSC as low, moderate and vigorous according to the international physical activity questionnaire (IPAQ).

**Table S2.** Determination of the number of clusters in the SDPP and MCS studies

|  | **Number of clusters (k)** | | | | | | | | |
| --- | --- | --- | --- | --- | --- | --- | --- | --- | --- |
| Cohort | 1 | 2 | 3 | 4 | 5 | **6** | 7 | 8 | 9 |
| SDPP | -0.059 | -0.033 | -0.012 | -0.016 | -0.010 | **0.000** | 0.004 | -0.006 | -0.003 |
| MSC | -0.049 | -0.026 | -0.001 | -0.007 | -0.008 | **0.001** | 0.004 | -0.003 | -0.000 |

Comparisons of different numbers of clusters according to equation (2). The optimal number of clusters is six in both data sets.

**Table S3.** Mean Jaccard similarity of the cluster categories in the SDPP and MSC studies.

| **Clusters** | **SDPP** | **MSC** |
| --- | --- | --- |
| **Very low-risk (VLR)** | 95.9% | 91.5% |
| **Low-risk low beta cell function (LRLB)** | 94.1% | 88.9% |
| **Low-risk high beta cell function (LRHB)** | 92.7% | 88.3% |
| **High-risk high blood pressure (HRHBP)** | 88.5% | 87.4% |
| **High-risk beta cell failure (HRBF)** | 91.4% | 95.1% |
| **High-risk insulin resistance (HRIR)** | 94.5% | 89.8% |

Jaccard similarity was estimated by reiterating the cluster algorithm using 1,000 bootstrap samples in each study. The index measures the magnitude of concordance between different iterations. Clusters with a coefficient greater than 75% are considered stable.

**Table S4.** Pairwise comparisons of the association between cluster membership and incidence of type 2 diabetes

| **SDPP** | **VLR** | **LRLB** | **LRHB** | **HRHBP** | **HRBF** |
| --- | --- | --- | --- | --- | --- |
| **LRLB** | 1.89^***^  (1.24, 2.87) | - | - | - | - |
| **LRHB** | 2.60^***^  (1.70, 3.97) | 1.38  (0.95, 1.99) | - | - | - |
| **HRHB** | 6.20^***^  (4.14, 9.29) | 3.28^***^  (2.34, 4.61) | 2.39^***^  (1.68, 3.39) | - | - |
| **HRHBP** | 8.71^***^  (6.05, 12.56) | 4.62^***^  (3.44, 6.20) | 3.35^***^  (2.47, 4.55) | 1.41^**^  (1.07, 1.85) | - |
| **HRIR** | 14.06^***^  (9.46, 20.88) | 7.45^***^  (5.35, 10.37) | 5.41^***^  (3.86, 7.58) | 2.27^***^  (1.66, 3.10) | 1.61^***^  (1.24, 2.09) |
| **MSC** | **VLR** | **LRLB** | **LRHB** | **HRHBP** | **HRBF** |
| **LRLB** | 2.11  (0.63, 7.08) | - | - | - | - |
| **LRHB** | 1.72^***^  (1.44, 2.06) | 0.82  (0.21, 3.23) | - | - | - |
| **HRHBP** | 5.63^***^  (2.12, 14.95) | 2.66^***^  (1.82, 3.89) | 3.27^*^  (1.03, 10.33) | - | - |
| **HRBF** | 6.71^***^  (2.78, 16.17) | 3.17^***^  (2.07, 4.86) | 3.89^**^  (1.35, 11.20) | 1.19^**^  (1.04, 1.36) | - |
| **HRIR** | 7.64^***^  (2.12, 27.52) | 3.62^***^  (2.65, 4.92) | 4.43^*^  (1.03, 19.00) | 1.36  (0.95, 1.93) | 1.14  (0.75, 1.73) |

SDPP: Stockholm diabetes prevention program, MSC: Metabolic syndrome cohort, VLR: Very low-risk cluster, LRHB: Low-risk high beta cell function cluster, LRLB: Low-risk low beta cell function cluster, HRHBP: High-risk high blood pressure cluster, HRBF: high-risk beta cell failure cluster and HRIR: High-risk insulin resistance cluster. Bonferroni correction was used to adjust for multiple comparison. ^*^: p-value <0.05, ^**^: p-value <0.01, ^***^: p-value <0.001.

**Table S5.** Accuracy of categories of prediabetes to predict type 2 diabetes in the SDPP study

|  | **ADA** | | | **WHO** | | |
| --- | --- | --- | --- | --- | --- | --- |
|  | **IFG** | **IGT** | **IFT+IGT** | **IFG** | **IGT** | **IFT+IGT** |
| **Sensitivity % (95%CI)** | 15.3%  (13.4%, 17.4%) | 9.3%  (7.8%, 10.9%) | 6.6%  (5.4%, 8.1%) | 5.2%  (4.1%, 6.6%) | 13.1%  (11.4%, 15.1%) | 2.8%  (2.0%, 3.8%) |
| **Specificity % (95%CI)** | 97.0%  (96.5%, 97.4%) | 98.5%  (98.2%, 98.8%) | 99.7%  (99.5%, 99.8%) | 99.4%  (99.2%, 99.6%) | 98.3%  (98.0%, 98.6%) | 99.9%  (99.8%, 100%) |
| **Area under the curve (95%CI)** | 0.56 (0.55 0.57) | 0.54 (0.53, 0.55) | 0.53 (0.52, 0.54) | 0.52 (0.52, 0.53) | 0.56 (0.55, 0.57) | 0.51 (0.51, 0.52) |
| **Positive predictive value % (95%CI)** | 51.1%  (46.1%, 56.1%) | 56.9%  (50.0%, 63.6%) | 82.4%  (73.9%, 89.1%) | 65.4%  (55.6%, 74.4%) | 61.8%  (55.8%, 67.4%) | 90.2%  (76.9%, 97.3%) |
| **Negative predictive value % (95%CI)** | 84.7%  (83.8%, 85.5%) | 84.0%  (83.1%, 84.8%) | 83.8%  (82.9%, 84.6%) | 83.5%  (82.7%, 84.3%) | 84.5%  (83.7%, 85.3%) | 83.2%  (82.4%, 84.1%) |

Measures of predictive accuracy of the different prediabetes states estimated during the baseline oral glucose tolerance test in the SDPP study. IFG: Impaired fasting glucose, IGT: Impaired glucose tolerance.

**Table S6.** Accuracy of individual clusters to predict type 2 diabetes in the SDPP study

|  | **VLR** | **LRHB** | **LRLB** | **HRHBP** | **HRBF** | **HRIR** |
| --- | --- | --- | --- | --- | --- | --- |
| **Sensitivity % (95%CI)** | 6.7%  (5.4%, 8.2%) | 11.5%  (9.77%, 13.4%) | 11.7%  (10.0%, 13.7%) | 15.6%  (13.6%, 17.7%) | 34.1%  (31.4%, 36.8%) | 20.4%  (18.2%, 22.8%) |
| **Specificity % (95%CI)** | 70.3%  (69.1%, 71.4%) | 74.7%  (73.6%, 75.8%) | 82.1%  (81.1%, 83.0%) | 91.2%  (90.4%, 91.9%) | 86.7%  (85.8%, 87.5%) | 95.1%  (94.5%, 95.6%) |
| **Area under the curve (95%CI)** | 0.39  (0.38, 0.40) | 0.43  (0.42, 0.44) | 0.47  (0.46, 0.48) | 0.53  (0.52, 0.54) | 0.60  (0.59, 0.62) | 0.58  (0.57, 0.59) |
| **Positive predictive value % (95%CI)** | 4.4%  (3.5%, 5.4%) | 8.4%  (7.12%, 9.83%) | 11.7%  (9.94%, 13.6%) | 26.2%  (23.2%, 29.6%) | 34.0%  (31.4%, 36.8%) | 45.7%  (41.5%, 50.0%) |
| **Negative predictive value % (95%CI)** | 78.9%  (77.8%, 80.0%) | 80.7%  (79.6%, 81.7%) | 82.2%  (81.2%, 83.1%) | 84.3%  (83.3%, 85.1%) | 86.7%  (85.8%, 87.5%) | 85.5%  (84.7%, 86.4%) |

Measures of predictive accuracy of the baseline cluster categories in the SDPP study. VLR: Very low-risk cluster, LRHB: Low-risk high beta cell function cluster, LRLB: Low-risk low beta cell function cluster, HRHBP: High-risk high blood pressure cluster, HRBF: high-risk beta cell failure cluster and HRIR: High-risk insulin resistance cluster.

**Table S7.** Accuracy of prediabetes and high-risk clusters to predict type 2 diabetes in the SDPP study

|  | **High-risk clusters** | **Prediabetes ADA** | **Prediabetes WHO** |
| --- | --- | --- | --- |
| **Sensitivity %(95%CI)** | 70.1% (67.4%, 72.6%) | 29.9% (27.4%, 32.6%) | 19.5% (17.3%, 21.8%) |
| **Specificity %(95%CI)** | 72.9%, (71.8%, 74.0%) | 95.3% (94.8%, 95.9%) | 97.8% (97.4%, 98.2%) |
| **Area under the curve (95%CI)** | 0.71 (0.70, 0.73) | 0.63 (0.61, 0.64) | 0.59 (0.58, 0.60) |
| **C-index (95%CI)** | 0.70 (0.68, 0.71) | 0.63 (0.61, 0.64) | 0.59 (0.58, 0.60) |
| **Positive predictive value %(95%CI)** | 34.3% (32.5%, 36.2%) | 56.5% (52.6%, 60.3%) | 64.4% (59.3%, 69.3%) |
| **Negative predictive value %(95%CI)** | 92.3% (91.6%, 93.1%) | 87.1% (86.3%, 87.9%) | 85.8% (84.9%, 86.6%) |

The predictive accuracy of prediabetes (any definition) and a high-risk cluster at baseline in the SDPP study. The high-risk clusters include the High-risk high blood pressure (HRHBP), the High-risk beta cell failure (HRBF) and the High-risk insulin resistance (HRIR) clusters.

**Table S8.** Intrarater reliability of clusters and prediabetes in the SDPP study

|  |  |  | **ADA** |  | **WHO** |  |
| --- | --- | --- | --- | --- | --- | --- |
|  | **VLR, LRLB, LRHB, HRHBP, HRBF, HRIR** | **High-risk clusters** | **NGT, IFG, IGT, IFG&IGT** | **Any Prediabetes** | **NGT, IFG, IGT, IFG&IGT** | **Any Prediabetes** |
| **Percent agreement** | 39.0% (37.9%, 40.2%) | 73.5% (72.4%, 74.6%) | 72.9% (71.7%, 74.1%) | 74.4% (73.3%,75.6%) | 85.1% (84.1%, 86.1%) | 85.6% (84.6%, 86.6%) |
| **Kappa** | 0.25 (0.23, 0.26) | 0.41 (0.38, 0.43) | 0.22 (0.20, 0.24) | 0.23 (0.21, 0.26) | 0.22 (0.19, 0.23) | 0.23 (0.20, 0.26) |
| **Gwet AC1** | 0.27 (0.26, 0.29) | 0.52 (0.50, 0.55) | 0.69 (0.68, 0.71) | 0.63 (0.60, 0.65) | 0.84 (0.83, 0.85) | 0.82 (0.81, 0.84) |

VLR: very low-risk, LRLB: low-risk low β-cell function, LRHB: low-risk high β-cell function, HRHBP: high-risk high blood pressure, HRBF: high-risk beta failure, HRIR: high-risk insulin resistance, NGT: normal glucose tolerance, IFG: impaired fasting glucose, IGT: impaired glucose tolerance.

Intrarater reliability indexes were estimated using the baseline, 10 year and 20 years follow-ups of the SDPP study. We estimated the coefficients using the six different clusters and a binary indicator dividing them into low-risk cluster and high-risk clusters. Similarly, for prediabetes, we estimated agreement using the different prediabetic states (IFG, IGT or both) and then as a binary measure combining all definitions.
