# Supplementary material for: Characterization of data-driven clusters in diabetes-free adults and their utility for risk stratification of type 2 diabetes
Source: BMC Med. 2022 Oct 18;20:356. doi: 10.1186/s12916-022-02551-6 (PMC9578256; doi:10.1186/s12916-022-02551-6)
Supplement: Supplementary file 2 — Additional file 2: Figure S1. Flow chart of the Stockholm Diabetes Preventive Program (SDPP). Figure S2. Flow chart of The Metabolic Syndrome Cohort (MSC). Figure S3. Determination of the number of clusters using Gap Statistic and within clusters sum of squares. Figure S4. Visualization of the clusters in SDPP (panel a) and MSC (panel b) using dimension reduction with u-MAP. Figure S5. Heatmap of the distribution of continuous variables by cohort. Figure S6. Box plots of the continuous variables used for cluster analysis, comparison between the SDPP and MSC cohorts. Figure S7. Baseline categorization as prediabetes and high-risk clusters and cases of type 2 diabetes in the SDPP study. Figure S8. Patterns of transition of prediabetes between the baseline, 10-year and 20-year follow-ups of the SDPP cohort. [file 12916_2022_2551_MOESM2_ESM.docx]

Characterization of data-driven clusters in diabetes-free adults and their utility for risk stratification of type 2 diabetes

Diego Yacaman Mendez^a,b^, Minhao Zhou^b^, Ylva Trolle Lagerros^c,d^, Donaji V. Gómez Velasco^e^, Per Tynelius^a,b^, Hrafnhildur Gudjonsdottir^a,b^, Antonio Ponce de Leon^b^, Katarina Eeg-Olofsson^f^ Claes-Göran Östenson, Boel Brynedal^a,b^, Carlos A. Aguilar Salinas^e^, David Ebbevi^a,b^, Anton Lager^a,b^

^a^ Department of Global Public Health, Karolinska Institutet. Stockholm, Sweden.

^b^ Centre for Epidemiology and Community Medicine (CES), Stockholm Health Care Services, Stockholm, Sweden.

^c^ Obesity Centre, Academic Specialist Centre, Stockholm Health Care Services, Stockholm, Sweden.

^d^ Unit of Clinical Epidemiology, Department of Medicine, Karolinska Institutet, Stockholm, Sweden.

e Unidad de Investigación de Enfermedades Metabólicas, Instituto Nacional de Ciencias Médicas y Nutrición “Salvador Zubirán”, Mexico City, Mexico.

f Department of Medicine, Sahlgrenska Academy, University of Gothenburg, Gothenburg, Sweden.

Correspondence to: Diego Yacaman-Mendez MD, MSc.

Department of Global Public Health, Karolinska Institutet, Stockholm, Sweden. SE-171 77.

Email address: diego.yacaman.mendez@ki.se

Declarations of interest: none.

**ADDITIONAL FILE 2: Supplementary figures**

**Figure S1.** Flow chart of the Stockholm Diabetes Preventive Program (SDPP).

Baseline clinical examination

n=7,948

Data available for cluster analysis at baseline:

n=7,317

1992–1998

Regional healthcare registry (VAL) and National Diabetes Registry (NDR)

N=7,317

2002–2006

10-year follow-up

n=5,327

20-year follow-up

n=3,987

All visits: 3,379

Baseline and 20y: 608

2014–2017

Type 1 diabetes: 17

Death: 375

Missing data: 438

Outliers: 78

Diabetes at baseline: 115

The data available for determining the clusters at baseline, the main survival analysis and the accuracy included 7,317 participants. The outcome was available for all participants who attended the baseline examination via the Regional healthcare Registry of Stockholm (VAL) and the National Diabetes Registry of Sweden (NDR).

To estimate the long-term stability via the intrarater agreement, data from 3,379 participants who attended all follow-ups was available.

**Figure S2.** Flow chart of The Metabolic Syndrome Cohort (MSC).

Age < 30 or > 60:at baseline: 1,839

Baseline examination

N=9,637

Follow-up visit

n=2,332

Data available for cluster analysis at baseline

n=3,832

2006–2009

2009–2014

n=7,798

Missing data: 3,932

Sex

Family history: 2,016

Education: 69

Fasting glucose: 1

Fasting Insulin: 95

Blood pressure: 1,735

BMI: 16

Outliers: 34

Lost to follow-up: 1,500

The MSC study was used as a replication sample to evaluate the reproducibility and external validity of the cluster categories. A selection of 2,332 participants at similar age groups and with complete data of the variables used in the cluster analysis was used in the current study.

**Figure S3**. Determination of the number of clusters using Gap Statistic and within clusters sum of squares


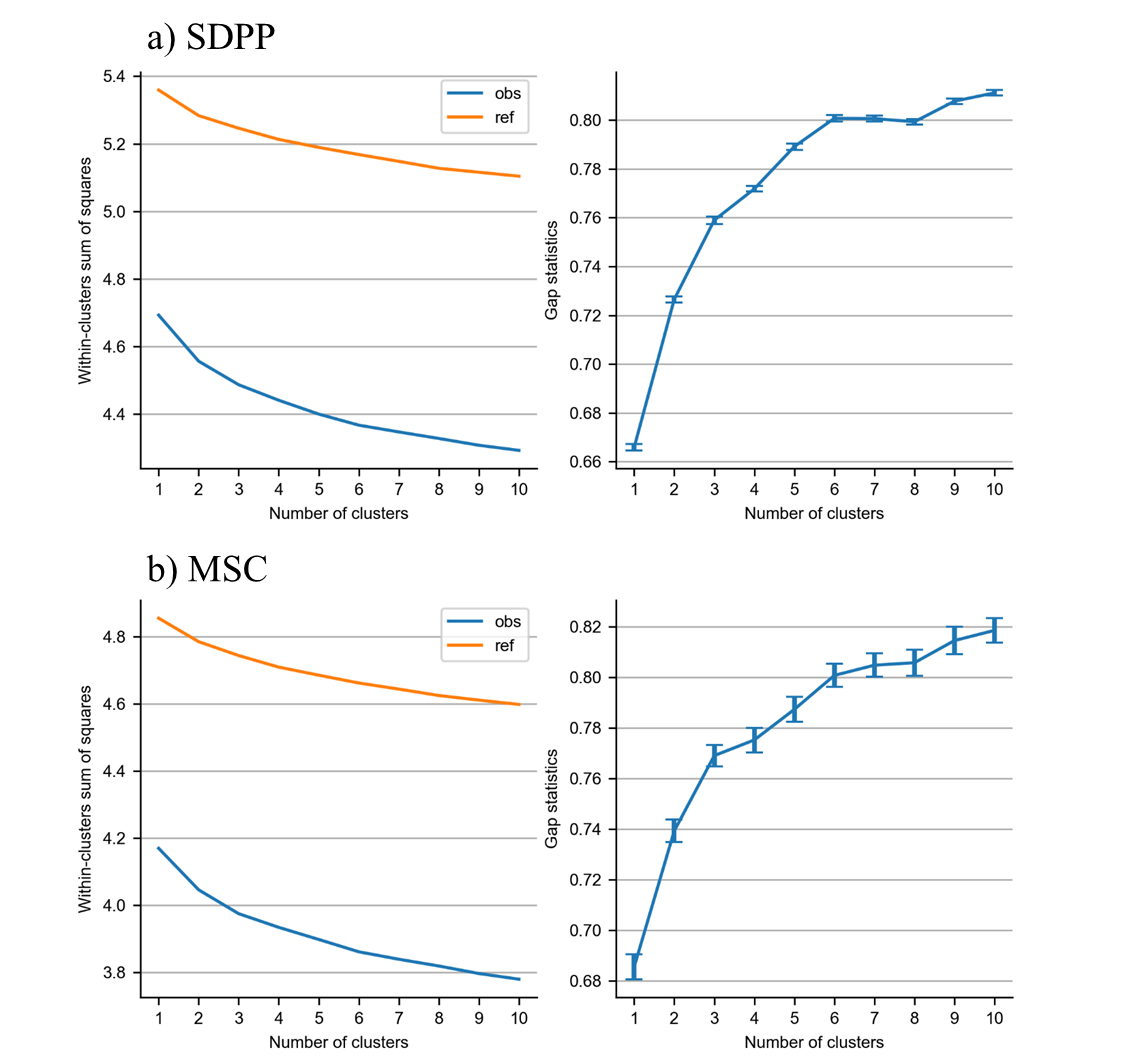


Graphic representation of the within sum of squares in the observed and reference populations (left), and of the Gap statistic (right) at increasing number of clusters (x-axis) in the Stockholm Diabetes Prevention Program (SDPP) and Metabolic Syndrome Cohort (MSC).

**Figure S4-**Visualization of cluster distributions using u-MAP


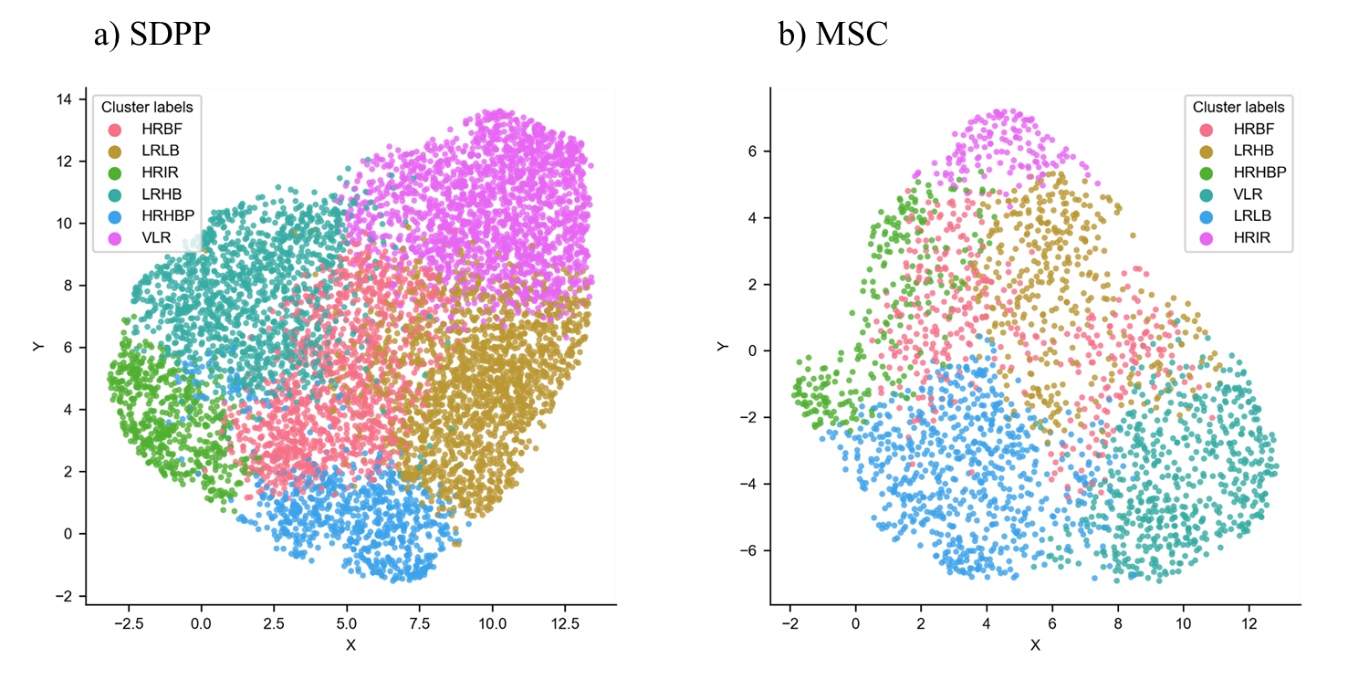


Visualization of the clusters in SDPP (panel a) and MSC (panel b) using dimension reduction with u-MAP. The distance matrix was pre-computed by using K-prototype dissimilarity. Interactive 3D visualization could be viewed at  *https://sdpp-data-driven-clusters.herokuapp.com/umap*.

**Figure S5.** Heatmap of the distribution of continuous variables by cohort


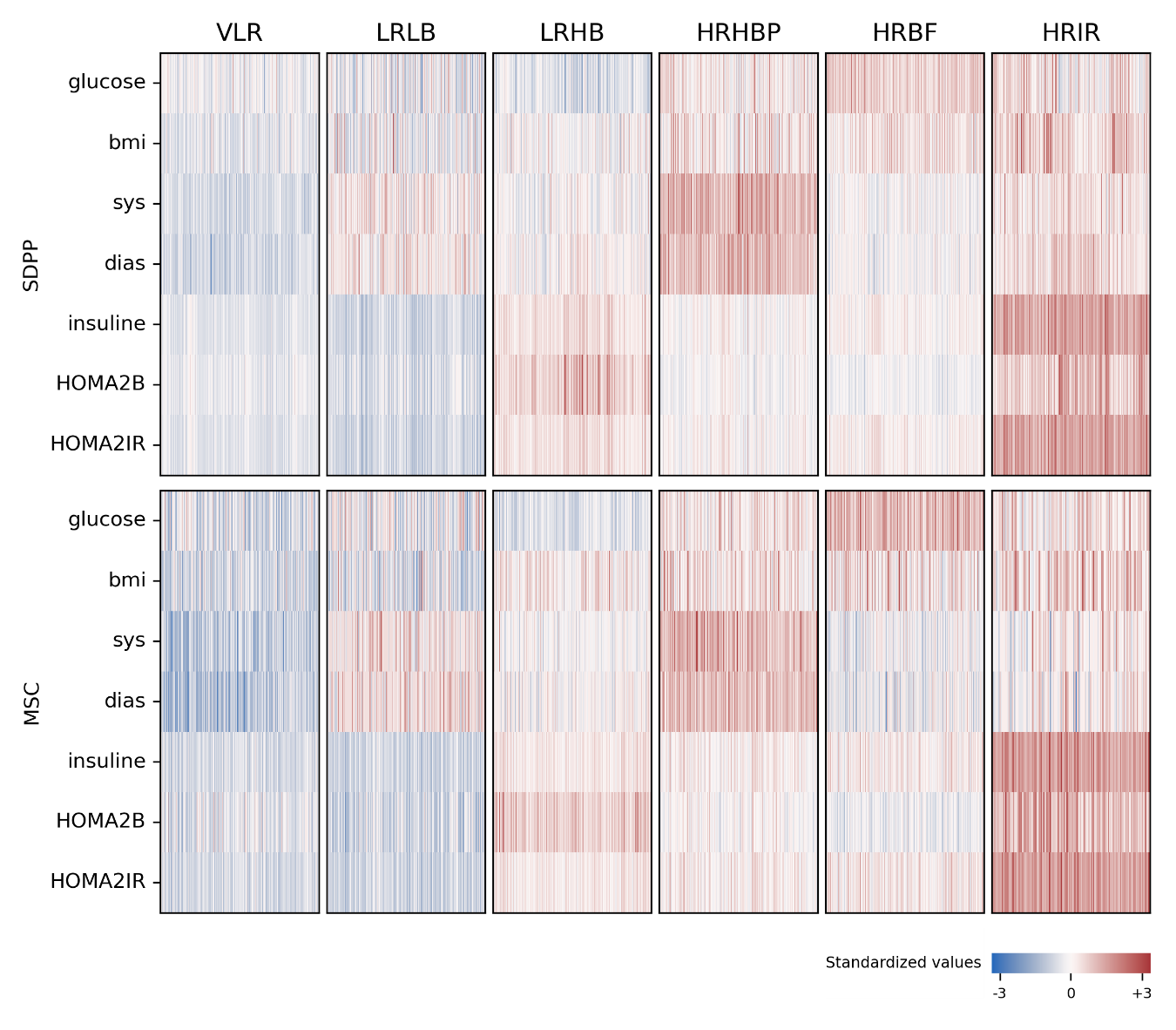


SDPP: Stockholm Diabetes Prevention Program, MSC: Metabolic Syndrome Cohort.

**Figure S6.** Continuous variables used for cluster analysis, comparison between the SDPP and MSC cohort.


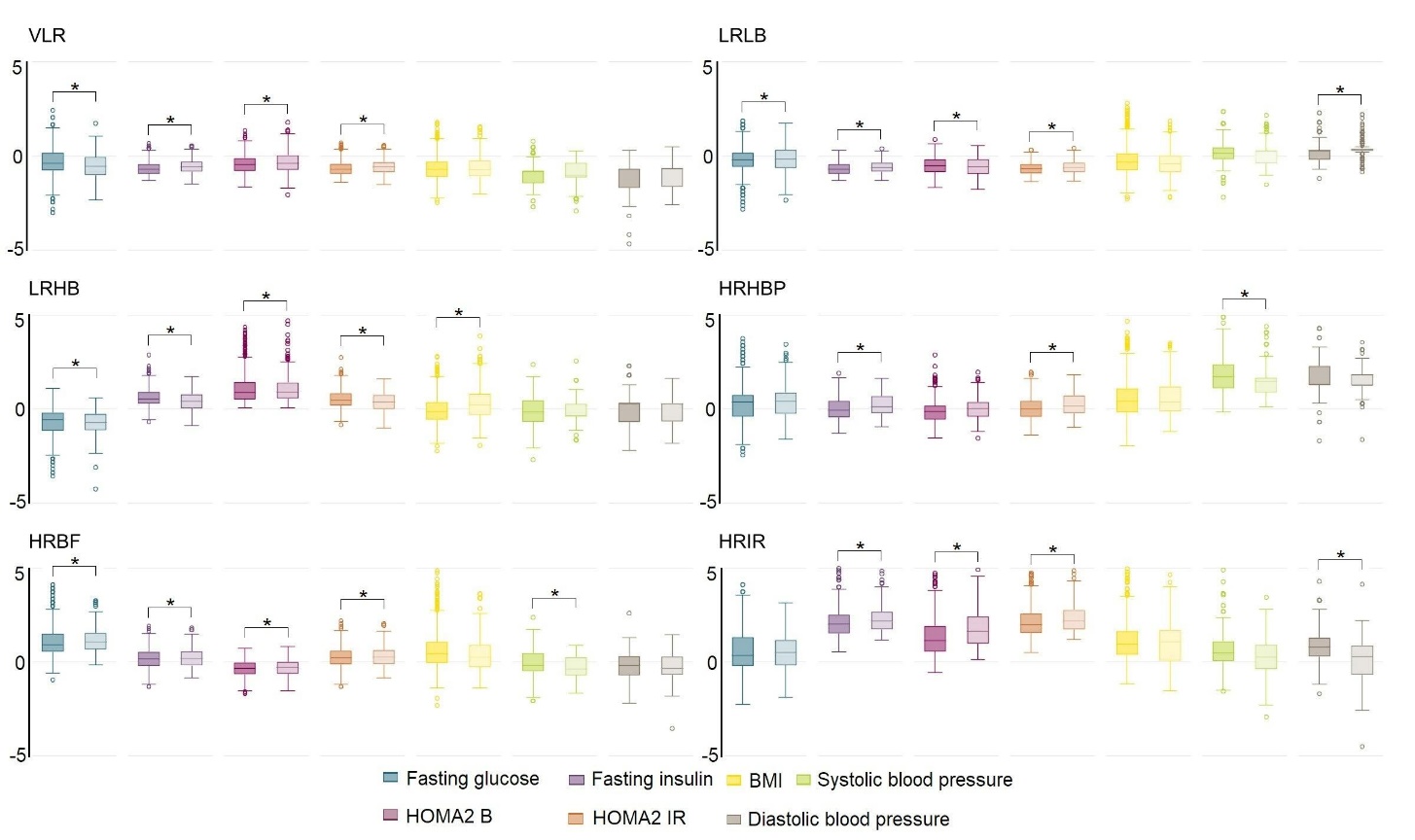


Values of the Stockholm diabetes prevention program (SDPP) are presented in full color and values of the Metabolic Syndrome Cohort (MSC) in translucent colors. Comparisons were done using t-test, Bonferroni correction was used to adjust for multiple comparison. ^*^: p-value <0.05. VLR: Very low risk cluster, LRHB: Low risk high beta cell function cluster, LRLB: Low risk low beta cell function cluster, HRHBP: High risk high blood pressure cluster, HRBF: high risk beta cell failure cluster and HRIR: High risk insulin resistance cluster.

**Figure S7.** Baseline categorization as prediabetes and high-risk clusters and cases of type 2 diabetes in the SDPP study

The y axis indicates the number of participants in each category, while the percentages inside the bars represent the within category percentages.

Although a large proportion of those categorized as having prediabetes at baseline developed type 2 diabetes during the study follow-up, the absolute number of participants who had a normal glucose tolerance at baseline and developed type 2 diabetes later on is much larger. In contrast, high risk clusters at baseline captured most cases of type 2 diabetes.

**Figure S8.** Transition plot of categories of prediabetes in the SDPP cohort


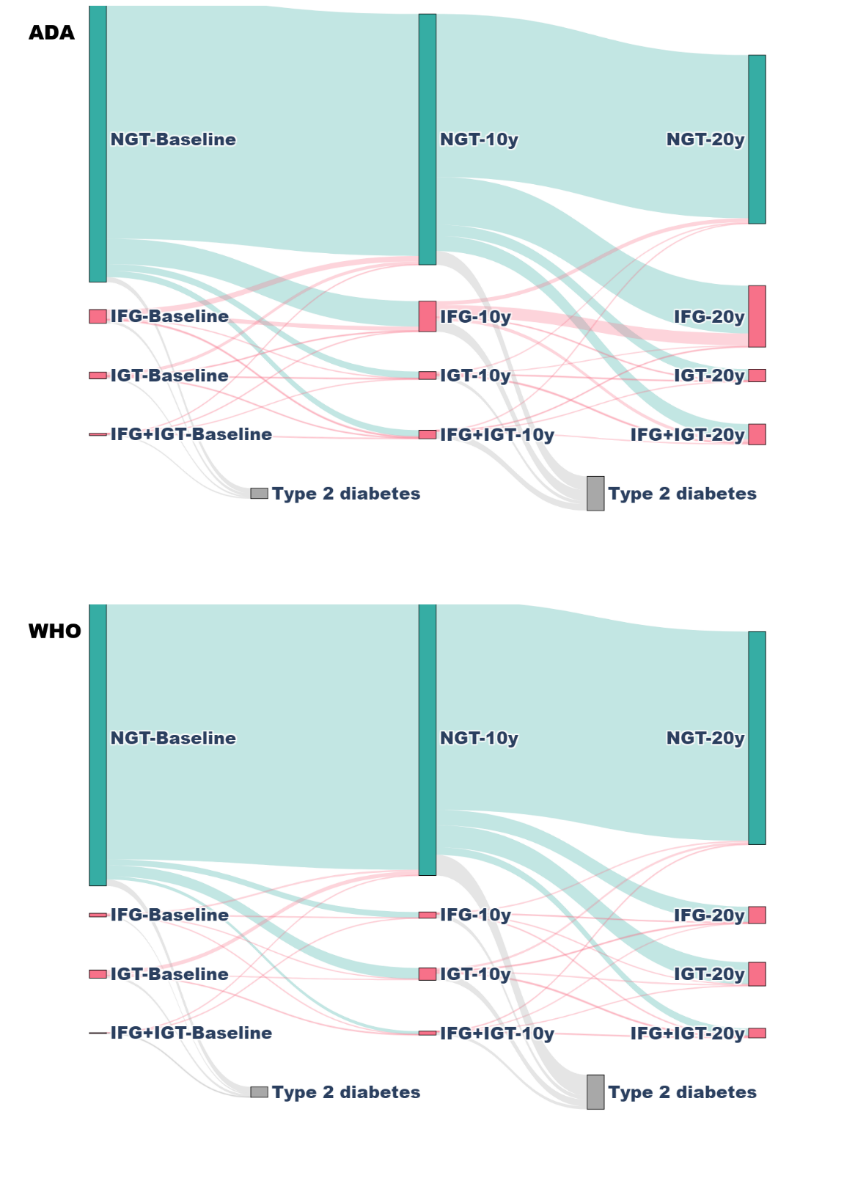


Patterns of transition of prediabetes between the baseline, 10-year and 20-year follow-ups of the SDPP cohort. The thickness of the line represents the proportion of individuals at each time-point. Prediabetes wass asserted based on fasting and 2-hour glucose levels. ADA: American Diabetes Association, WHO: World health organization, NGT: Normal glucose tolerance (blue), IFG: Impaired fasting glucose, IGT: Impaired glucose tolerance, IFG+IGT: Impaired fasting glucose and impaired glucose tolerance (All subtypes of prediabetes are marked red)
