# Supplementary material for: Characterization of data-driven clusters in diabetes-free adults and their utility for risk stratification of type 2 diabetes
Source: BMC Med. 2022 Oct 18;20:356. doi: 10.1186/s12916-022-02551-6 (PMC9578256; doi:10.1186/s12916-022-02551-6)
Supplement: Supplementary file 3 — Additional file 3. Detailed description of methods for cluster analysis and computer code (Stata and Python). [file 12916_2022_2551_MOESM3_ESM.docx]

Characterization of data-driven clusters in diabetes-free adults and their utility for risk stratification of type 2 diabetes

Diego Yacaman Mendez^a,b^, Minhao Zhou^b^, Ylva Trolle Lagerros^c,d^, Donaji V. Gómez Velasco^e^, Per Tynelius^a,b^, Hrafnhildur Gudjonsdottir^a,b^, Antonio Ponce de Leon^b^, Katarina Eeg-Olofsson^f^ Claes-Göran Östenson, Boel Brynedal^a,b^, Carlos A. Aguilar Salinas^e^, David Ebbevi^a,b^, Anton Lager^a,b^

^a^ Department of Global Public Health, Karolinska Institutet. Stockholm, Sweden.

^b^ Centre for Epidemiology and Community Medicine (CES), Stockholm Health Care Services, Stockholm, Sweden.

^c^ Obesity Centre, Academic Specialist Centre, Stockholm Health Care Services, Stockholm, Sweden.

^d^ Unit of Clinical Epidemiology, Department of Medicine, Karolinska Institutet, Stockholm, Sweden.

e Unidad de Investigación de Enfermedades Metabólicas, Instituto Nacional de Ciencias Médicas y Nutrición “Salvador Zubirán”, Mexico City, Mexico.

f Department of Medicine, Sahlgrenska Academy, University of Gothenburg, Gothenburg, Sweden.

Correspondence to: Diego Yacaman-Mendez MD, MSc.

Department of Global Public Health, Karolinska Institutet, Stockholm, Sweden. SE-171 77.

Email address: diego.yacaman.mendez@ki.se

Declarations of interest: none

# ADDITIONAL FILE 3: Methods for cluster analysis and computer code

# Cluster analysis

## K-prototype

The aim of cluster analysis is to divide a complex data set into homogeneous subgroups, under the assumption that they represent distinctive underlying populations. There are several methods for cluster analysis, in the present study we used k-prototype, a partitioning method that has the main advantage of accommodating both continuous and categorical data.

K-prototype is a generalization of k-means, a clustering algorithm useful for continuous data, and k-modes, used for categorical data. The distance between individual data points is measured using a weighted dissimilarity measure, combining the Euclidean distance used in k-means with the number of mismatches of categorical data, the dissimilarity measure used in k-modes. Formally, the dissimilarity between two objects X and Y whose attributes A*r* 1, A*r* 2,….,A*r* *p* are numeric and A*c p+1*,…., A*c m* are categorical is defined as

$$\begin{aligned} d\left( X,Y \right)=\sum_{j=1}^{p} \left( x_{j}-y_{j} \right)^{2}+\gamma\sum_{j=p+1}^{m} \delta\left( x_{j}, y_{j} \right)\#\left( 1 \right) \end{aligned}$$

Where the first term represents the Euclidean distance of the continuous attributes, and $\delta\left( x_{j}, y_{j} \right)$ represents the number of mismatches of categories between the two objects. The weight γ, is a pre-defined parameter indicating how much the measurement favors the categorical attributes. Huang suggested that a suitable γ lies between 1/3*σ* and 2/3*σ* where *σ* is the average standard deviation of numeric attributes. Thus, in this study, γ was set to 0.5.

Next, a reiterative process is used to determine the function P (or cost function), which represents the optimal partition (W) of a set of observations (Q) into a predefined number of clusters (*k*) that minimizes the within cluster variability starting from random cluster centers (*v*). In other words, finding the best way to divide the observation into homogeneous clusters.

$$\begin{aligned} P\left( W,v;Q \right)=\sum_{l=1}^{k} \sum_{i=1}^{n} w_{i,l}d\left( X_{i},Q_{l} \right)\#(2 \end{aligned})$$

Where v= (v1,v2,…,vk) is a vector of cluster centers or centroids that are assigned at random, W represents an *n×k* partition matrix, {*Q*=Q1, Q2,..., Q} a set of objects in the same cluster, and $d\left( X_{i},Q_{l} \right)$ represents the distance measured according to (1).

## Determination of the number of clusters

Determining the optimal number of clusters is one of the main challenges of cluster analysis. As mentioned previously, partitioning clustering algorithms such as k-prototype require the number of clusters (*k*) as a predefined parameter.

In this study, we used the Gap statistic. A quantitative method proposed by Tibshirani and collaborators that uses the within cluster variability of *k* numbers of clusters compared to a reference distribution for which we know there are no clusters. The point in which the difference (or gap) between the observed and expected variability, measured as the logarithm of the within sum of squares, is maximized represents the most optimal number of underlying clusters in a particular data set (ref).

The Gap is given by the formula:

$$\begin{aligned} {Gap}_{\left( k \right)}= E_{n}^{*}\left\{ \log\left( W_{k} \right) \right\}-\log\left( W_{k} \right) \#(3) \end{aligned}$$

Where $E_{n}^{*}$ is the expected value from the reference distribution and $W_{k}$ the within cluster sum of squares.

The optimal number of clusters is then determined by comparing the ${Gap}_{\left( k \right)}$to ${Gap}_{\left( k+1 \right)}$. The *k* at which this difference is ≥ 0 represents the point at which increasing number of clusters does not further significantly reduce the within cluster variability.

$$\begin{aligned} {Gap}_{k}-{Gap}_{k+1}+s_{k+1}\geq0 \#(4) \end{aligned}$$

$s_{k}$denotes the standard distribution of ${Gap}_{k}$.

Finally, it is important to assess if the cluster algorithm would yield similar results every time it is estimated in a random sample coming from the same population. This is known as cluster-wise stability, a low stability might indicate a spurious cluster due to noise in the data set.

We calculated the mean Jaccard similarity coefficient between 1,000 bootstrap samples to assess cluster-wise stability. The Jaccard similarity coefficient It is defined as the division between the number of clusters common in the original data set and the bootstrapped sample and the number of components that differ in the two sets (ref).

$$\begin{aligned} J\left( A,B \right)=\frac{\left| A\cap B \right|}{\left| A\cup B \right|} \#(5) \end{aligned}$$

A represents the clusters in the original data set, while B the cluster category in the bootstrap sample for a given individual. $A\cap B$Is the intersection between A,B. $A\cup B$ represents the union between A,B.

# Code

## Cluster analysis: Stata/Phyton.

#### Import necessary packages

import pandas as pd
import numpy as np
from src_for_submission import * #available from Github repository
from datetime import datetime
import pickle
from kpplus import KPrototypes_plus
import importlib
import sys
import plotly.express as px
import plotly.graph_objects as go
from PIL import Image
import io
import os

#### Gap statistcs

sdpp_gap_return = gap_statistics_k_prototype(data = df_sdpp_analysis, #get gap statistics
 categorical_column_id = [0,1,2],
 max_n_cluster = 10,
 n_init = 10,
 n_ref = 10,
 gamma = 0.5)

gap_plot(gap_return = sdpp_gap_return, #plot gap statistics
 file_name = 'sdpp_gap_statistics_%s'%(str(timenow_strf)))

print(sdpp_gap_return[0][:-1] - sdpp_gap_return[0][1:] + sdpp_gap_return[3][1:]) #the optimal k should be the first k that with non negative value

#### Create and save cluster labels

sdpp_mdl = KPrototypes_plus(n_clusters = 6, n_init = 10, gamma = 0.5) #Initialize the model
sdpp_mdl.fit_predict(X = df_analysis, categorical = [0,1,2])
cluster_labels = mdl.labels_
init_prototype = mdl.init_prototype_
with open('init_prototype_%s.pickle'%str(timenow_strf), 'wb') as f: #save initiation_prototype as pickle file
 pickle.dump(init_prototype, f)
df['cluster_labels'] = cluster_labels + 1 #change cluster labels start from 1 instead of 0
df[['id', 'idnr', 'cluster_labels7']].to_csv('clusterlabels_sdpp_noage_6cl_%s.csv'%str(timenow_strf), encoding = 'latin1') #save sdpp cluster label as csv file

#### Jaccard stability

cluster_labels = pd.read_stata('analysis_sdpp_6cl_210930.dta').dropna(subset = ['cluster_labels7'])['cluster_labels'].astype('str')
with open('init_prototype_210930.pickle', 'rb') as f:
 init_prototype = pickle.load(f)
jaccard_coefficient_same_init(data = df_analysis.values,
 categorical=[0,1,2],
 cluster_labels = sdpp_cluster_labels,
 initial_prototype = sdpp_init_prototype,
 number_of_repetation=1000,
 gamma = 0.5)

## Survival analysis, accuracy, and reliability (Stata 15)

### Survival analysis

* Set data for time to event analysis

stset agediag, id(id) failure(diab) enter(age1)

stdescribe

* Test proportional hazard assumption

eststo model: stcox ib3.cluster_labels7, vce(robust)

** Test

estat phtest , detail

** Log-log plot of survival

stphplot , by(cluster_labels)

** Kaplan -Meier versus predicted survival

stcoxkm, by(cluster_labels)

* Person-time and incidence rates per cluster

stptime, by(cluster_labels7) per(1000)

* Fit unadjusted and adjusted Cox proportional hazard models

eststo clear

eststo model: stcox ib3.cluster_labels, vce(robust)

eststo mode2: stcox ib3.cluster_labels age1 i.gralh i.active1 i.gestdiab, vce(robust)

esttab, ci(%9.2f) b(%9.2f) eform

* Kaplan-Meier graph

sts graph, by(cluster_labels) tmax(79) xsize(6) noorigin legend(col(4)) legend(order(6 5 4 3 1 2)) title("SDPP") ytitle(Survival probability) xlabel(30 40 50 60 70 80) xtitle(Age)

*** Pairwise comparisons between individual clusters

qui stcox ib4.cluster_labels7 age1 i.gralh i.chronicdx i.active1 i.smoke11 , strata(bcohort)

pwcompare cluster_labels7, asobs effects mcompare(bonferroni) pformat(%9.2f) cformat(%9.2f)

### Accuracy

* Sensitivity, specificity, and area under the curve

Ssc install diagt

diagt diab pred1

diagt diab clustcat

* each cluster and each category of predeiabetes individually

tab cluster_labels, gen(cs)

foreach n of numlist 1/6 {

diagt diab cs`n'

}

tab ada041, gen(predclass) ## ada041= 1=normal OGTT, 2= IFG, 3 IGT, 4=IFG+IGT

foreach n of numlist 1/4 {

diagt diab predclass`n'

}

** Harsells C pred and clusters

** All clusters

qui stcox ib3.cluster_labels7, vce(robust)

estat concord

predict hr

generate invhr=1/hr

generate censind=1-_d if _st==1

somersd _t invhr if _st==1, cenind(censind) tdist transf(c)

somersd _t invhr if _st==1, cenind(censind) tdist transf(z)

** High-risk clusters

qui stcox i.clustcat , vce(robust)

estat concord

predict hr2

generate invhr2= 1/hr2

generate censind2=1-_d if _st==1

somersd _t invhr2 if _st==1, cenind(censind2) tdist transf(c)

somersd _t invhr2 if _st==1, cenind(censind2) tdist transf(z)

** prediabetes

qui stcox i.pred1 , vce(robust)

estat concord

predict hr1

generate invhr1=1/hr1

generate censind1=1-_d if _st==1

somersd _t invhr1 if _st==1, cenind(censind1) tdist transf(c)

somersd _t invhr1 if _st==1, cenind(censind1) tdist transf(z)

### Intrarater reliability

Ssc install kappaetc

kappaetc clust1 clust21 clust31, wgt(id) listwise

kappaetc cluster_labels_baseline cluster_labels_10year cluster_labels_20year, wgt(id) listwise

*

gen clustcat21= cscat2

replace clustcat21= clustcat1 if clustcat2==3

tab clustcat2 clustcat21

gen clustcat31= cscat3

replace clustcat31= clustcat2 if cluscat3==3

tab cluscat3 clustcat31

kappaetc cscat1 cscat2 cscat3, wgt(id) listwise

kappaetc cscat1 clustcat21 clustcat31, wgt(id) listwise

** prediabetes stable if going to diab

gen pred21= pred2

replace pred21= pred1 if clustcat2==3

tab pred21 pred2

gen pred31= pred3

replace pred31= pred2 if cluscat3==3

tab pred31 pred3

kappaetc pred1 pred21 pred31 if clustcat1!=., wgt(id) listwise

gen predcat21= predcat2

replace predcat2=predcat1 if clustcat2==3

tab predcat21

gen predcat31= predcat3

replace predcat31=predcat2 if cluscat3==3

tab predcat31

kappaetc predcat1 predcat21 predcat31 if clustcat1!=., wgt(id) listwise
